# miR-155-5p upregulation ameliorates myocardial insulin resistance via mTOR signaling in chronic alcohol drinking rats

**DOI:** 10.7717/peerj.10920

**Published:** 2021-04-05

**Authors:** Zhaoping Li, Zhenzhen Hu, Yan Meng, Hongzhao Xu, Yali Wei, Deqiang Shen, Hao Bai, Huacai Yuan, Liyong Chen

**Affiliations:** 1 Department of Clinical Nutrition, Shandong Provincial Hospital Affiliated to Shandong First Medical University, Jinan, Shandong, China; 2 Department of Clinical Nutrition, Shandong Provincial Hospital, Cheeloo College of Medicine, Shandong University, Jinan, Shandong, China; 3 Department of Toxicology and Nutrition, School of Public Health, Cheeloo College of Medicine, Shandong University, Jinan, Shandong, China; 4 Department of Clinical Nutrition, Lianyungang Hospital Affiliated to Xuzhou Medical University, Lianyungang, Jiangsu, China; 5 Department of Epidemiology and Biostatistics, School of Public Health, Zhejiang University, Hangzhou, Zhejiang, China; 6 Department of Clinical Nutrition, Qingdao Municipal Hospital, Qingdao, Shandong, China

**Keywords:** Alcoholic cardiomyopathy, microRNA-155, Insulin resistance, mTOR signaling, Alcohol consumption

## Abstract

**Background:**

Chronic alcohol intake is associated with an increased risk of alcoholic cardiomyopathy, which may present with pathological changes such as myocardial insulin resistance, leading to ventricular dilation and cardiac dysfunction. Although a correlation between microRNA-155 (miR-155) and insulin signaling has been identified, the underlying mechanism has not been elucidated to date. The purpose of the study was to determine whether overexpression of miR-155-5p in vivo could ameliorate chronic alcohol-induced myocardial insulin resistance and cardiac dysfunction.

**Material and Methods:**

Wistar rats were fed with either alcohol or water for 20 weeks to establish chronic alcohol intakes model. Then the alcohol group were divided into three groups: model group, miRNA-155 group and AAV-NC group. Rats undergoing alcohol treatment were injected with AAV-miRNA-155 (adeno-associated virus 9) or its negative control AAV-NC, respectively. Gene expression was determined by real-time PCR, and protein expression was determined by western blot. Echocardiography was performed to assess terminal cardiac function. Insulin responsiveness was determined through the quantification of phosphorylated insulin receptor substrate 1 (ser 307) and phosphorylated insulin receptor (Tyr 1185) levels.

**Results:**

We found that cardiac function was attenuated in chronic alcohol intake rats, with an activated mammalian target of rapamycin (mTOR) signaling pathway, accompanied by an increase in p-IRS1(ser 307) and a decrease in p-IR (Tyr 1185) level in myocardial tissue. Also, alcohol drinking significantly up-regulated miR-155-5p level and its overexpression decreased p-IRS1 (ser 307) and increased p-IR (Tyr 1185) levels, and meanwhile inhibited the mTOR signaling pathway.

**Conclusion:**

miR-155-5p upregulation ameliorates myocardial insulin resistance via the mTOR signaling in chronic alcohol drinking rats. We propose that miR-155 may serve as a novel potential therapeutic target for alcoholic heart disease.

## Introduction

Alcoholic cardiomyopathy (ACM), which presents as a left ventricular dilated cardiomyopathy that leads to heart failure and increased cardiac mortality (Fernandez-Sola et al., 1994; Zhang et al., 2004), has become a major global health problem due to the high prevalence of alcohol abuse (Mirijello et al., 2017). Alcohol intake is an remote cause of nonischemic dilated cardiomyopathy (Gavazzi et al., 2000). Manthey et al. (2017) predicted that the death of ACM patients in China account for 5.7% (95% CI [3.4–9.0]%) of all cardiomyopathy related death. While, the 4-year mortality rate of patients with alcoholic cardiomyopathy is up to 50% (Skotzko et al., 2009). It is important to identify therapeutic interventions at preventing the development and progression of ACM.

Various pathological changes have been identified in myocardium during the occurrence and development of ACM including myocardial insulin resistance (Han et al., 2018). Clinical evidence has demonstrated insulin resistance is independently associated with left ventricular diastolic dysfunction (Dinh et al., 2010). The mammalian target of rapamycin (mTOR) is an evolutionarily conserved serine/threonine kinase, which is involved in various physiological processes, including cell metabolism, survival, and autophagy (Karar & Maity, 2011). Moreover, the mTOR signaling pathway regulates glucose metabolism and plays a crucial role in insulin resistance (Yoon, 2017). Overactivation of the mammalian target of rapamycin complex 1 (mTORC1) can lead to insulin resistance. Furthermore, mTORC1 generates an inhibitory feedback loop along with insulin receptor substrate proteins (Zoncu, Efeyan & Sabatini, 2011). Interestingly, mTOR can inhibit phosphatidylinositol 3-kinase (PI3K)/Akt by phosphorylating insulin receptor substrate-1(IRS-1) at Ser-636/639 in diabetic-mimicking conditions (Tzatsos, 2009).

MicroRNAs are a class of small non-coding RNA molecules that are involved in many biological processes including proliferation, differentiation, and apoptosis and regulate the development and progression of vascular damage (Zampetaki & Mayr, 2012). MicroRNA-155 (miR-155) directly modulates the expression of three members of the mTOR pathway Ras homolog enriched in brain (Rheb), rapamycin insensitive companion of mTOR (Rictor), and ribosomal protein S6 kinase B2 (S6K2) and affects cell proliferation and G1/S cell cycle progression. Furthermore, transfection with miR-155 decreases the expression of Rheb and mTOR, whereas inhibition of miR-155 plays a protective role in rats with ischemic stroke via phosphorylation of S6K through the Rheb/mTOR pathway (Xing et al., 2016). Yin et al. (2018) reported that miR-155 promotes endothelial cell autophagy by regulating the PI3K/Akt/mTOR pathway, which inhibits inflammation and exerts a preventive role against the development of atherosclerosis. In addition, miR-155 is also involved in the occurrence of epilepsy through PI3K/Akt/mTOR signaling pathway (Duan, Chen & Wang, 2018). These observations suggested miR-155 plays a crucial role in PI3K/Akt/mTOR signaling pathway. However, it is unknown whether miR-155 exerts a protective effect against ethanol-induced myocardial insulin resistance via the mTOR signaling pathway. Here, we investigated whether overexpression of miR-155-5p in vivo could ameliorate myocardial insulin sensitivity and cardiac function and explored the role of miR-155-5p regulated mTOR signaling pathway in chronic alcohol consumption induced heart dysfunction.

## Materials & Methods

### Animal and treatments

This study was performed at the Center for Animal Experiments of Shandong University and it was approved by the experimental animal ethics committee of Shandong University provincial hospital (No:2020-775). Specific pathogen-freemale Wistar rats (160–200 g) were purchased from Ji’nan Pengyue Animal Breeding Co., Ltd. (No. 37009200013609, certification number: 370181000009090, license number: SCXK [lu] 20140007). All animals were housed for a week under standard laboratory conditions with a 12 h light: 12 h dark cycle at 24 ± 1 °C to allow their adaptation and Wistar male rats then were divided into two groups: control group (*n* = 8) and model group (*n* = 24). Four animals were sacrificed for unknown reasons during the experiment. According to a previously described model for free-drinking (Holguin et al., 1998; Kryzhanovskii et al., 2017), the model group rats were provided 3% ethanol solution instead of water for 3 days, then 6% for 4 days, and finally 10% ethanol solution as the only source of fluid for 20 weeks; the mean daily ethanol consumption was 6.5–8.0 g/kg. After 20 weeks, model rats were randomly divided into three groups: The Model group, AAV-NC group and AAV-miR-155 group. To overexpress miR-155-5p, rats were injected with AAV-miR-155 two weeks after chronic alcohol intake. After a 14-day observation period, an ultrasonic cardiogram (UCG) was performed on the surviving animals. Two weeks later, after 12 h of food deprivation the rats were anesthetized, abdominal aortic blood, hearts were taken and blood samples were collected at 8 a.m.

### Design, synthesis and transfection with AAV- miR-155-5p

The adeno-associated virus pre-miR-155-5p (AAV-miR-155-5p, AAV 9) was purchased from OBiO Corporation (Shanghai, China). The rnomiR-155-Eco-Forward primer was: CGGCGGTTAATGCTAATTGTGAT, and the rno-miR-155-Bam-Reverse primer was GTGCAGGGTCCGAGGT. To evaluate the effect of miR-155-5p treatment in rats with alcohol consumption, a left thoracotomy was performed, and the pericardium was stripped to fully expose the heart. Animals were intramyocardially injected with AAV containing either an miR-155-5p precursor (*n* = 8) or an AAV-NC (negative control, *n* = 8) as previously reported protocol (Li et al., 2020a; Zhou, Fu & Chen, 2019). In short, 10% chloral hydrate (0.03 mL/kg) was administered for anesthetization and connected with a rodent ventilator to maintain artificial respiration. The skin was cut longitudinally 0.5 cm on the left side of the sternum. The fascia and intercostal muscles were separated passively and then the internal intercostal muscles at the obvious place of heart beat. Left pleural cavity was opened and then the pericardium. 100 µl (5.0 × 10^12^ V.G./ml) recombinant lentiviral solution was injected into the myocardium at 4 points 2 mm above and below the heart apex 0.5 mm from the long axis of the heart (25 µL for each site). After the procedure, the chest was closed. rats were injected with 0.5% bupivacaine for pain relief. After a 14-day observation period, an ultrasonic cardiogram (UCG) was performed on the surviving animals. All surgeries were performed under aseptic conditions, and rats were anesthetized using 10% chloral hydrate (0.03 mL/kg). Cardiac muscle tissue was collected 15 days after the surgery for quantitative polymerase chain reaction (q-PCR) and western blotting.

### Cardiac function monitoring

Cardiac geometry and function were evaluated using the DP-50 imaging system (Mindray, Shenzhen, China). Rats were anesthetized with 1.5% isoflurane. In M-mode images, we evaluated the left ventricular internal diastolic diameter (LVIDd), left ventricular end systolic diameter (LVIDs), left ventricular posterior wall depth (LVPWd), left ventricular posterior wall thickness at end-systole (LVPWs), left ventricular ejection fraction (LVEF), end-diastolic volume (EDV), stroke volume (SV), and fractional shortening (FS). All measurements were performed by the same observer who was blinded to the study group; all measurements were repeated thrice to ensure the reproducibility of the results.

### Oral glucose tolerance tests (OGTT)

Rats were fasted overnight (12 h) three days before sacrifice and determined blood glucose (BG) levels using a glucometer then received 20% glucose loading by gastric gavage (0.1 ml/10 g of body weight). Blood samples were collected from the tail vein at 30, 60 and 120 min after glucose loading. At the end of blood collection, rats were disinfected and hemostatic. Area under curve (AUC) was calculated according to the formula [1/4BG (0 min) + 1/2BG (30 min) + 3/4 BG (60 min) + 1/2 BG (120 min)] to evaluate glucose tolerance. Blood glucose was determined using an automatic glucose meter (Roche, Germany).

### Insulin sensitivity assessment

After 12 h of food deprivation, rats were anesthetized using 10% chloral hydrate (0.03 mL/kg), abdominal aortic blood samples then centrifuged at 3500G for 10 min for plasma collection. Plasma samples were stored at −80 °C until further use. Fasting blood glucose (FBG) was determined by Glucose Oxidase method, and fasting blood insulin (FINS) level was determined by radioimmunoassay to evaluate insulin sensitivity at body level by Homoeostasis model assessment-insulin resistance index (HOMA-IR). The formula for calculation is as follows: HOMA-IR = FBG × FINS/22.5. Insulin ELISA kit (Cloud-Clone, China) was used to detect serum insulin levels.

### Real-time PCR

Total RNA was prepared using RNAiso Plus (code no. 9108, Takara) according to the kit instructions. RNA purity and concentration were determined using a NanoDrop™ 2000 Microvolume Spectrophotometer (Thermo Fisher Scientific). Reverse transcription was performed utilizing a Revert Aid First Strand cDNA Synthesis Kit (#K1622, Thermo). The expression of Rheb, S6K2, and Rictor mRNA was quantified using primers designed by Wuhan Qinke Innovation Biotechnology Co., Ltd., on a 7300 Real-Time PCR System (Applied Biosystems). The expression of Rheb, S6K2, and Rictor in the samples were normalized to GAPDH as the control, and for miR-155-5p was normalized toU6. The primer sequences (5′–3′) and elementary procedure were showed in Tables 1 and 2.

### Western blot analysis

Samples of cardiac tissue protein were prepared using a protein Extraction Kit (Solarbio, Beijing, China). Protein concentration was determined using a bicinchoninic acid (BCA) Protein Assay kit (Solarbio, Beijing, China). The following primary antibodies were used in this study: anti-Rheb (Abcam, ab25873), anti-S6K2 (Cell Signaling, 14130), anti-Rictor (Cell Signaling, 2140), anti-phospho-IR (Abcam, ab62321), anti-phospho-IRS-1 (Ser 307) (Cell Signaling, 2381), anti-mTOR (phospho S2448) (Abcam, ab109268/R), anti-mTOR (Abcam, ab32028), and anti-GAPDH (ZSGB-BIO, TA-08/M). Protein samples were separated on 6% (for Rictor and p-IRS-1) or 12% (for S6K2, Rheb, and GAPDH) SDS-PAGE gels, and were transferred onto polyvinylidene fluoride (PVDF) membranes. The membranes were blocked with 5% skim milk, after which they were incubated with a 1:1000–1:2000 dilution of primary antibodies overnight at 4 °C. Subsequently, the membranes were washed and incubated with horseradish peroxidase-conjugated secondary antibodies (1:5000 dilution) for 1 h at room temperature (about 25 °C). The enhanced chemiluminescence (ECL) method was utilized to visualize the protein bands.

### Statistical analysis

Statistical analysis was performed using the SPSS 24.0 software (Chicago, IL, USA). All results are presented as means ± SD unless stated otherwise. The data were analysed using *t*-test. *P*-values lower than 0.05 were denoted as statistically significant.

## Results

### Myocardial insulin sensitivity were attenuated in chronic alcohol intake rats

**Figure 1:**
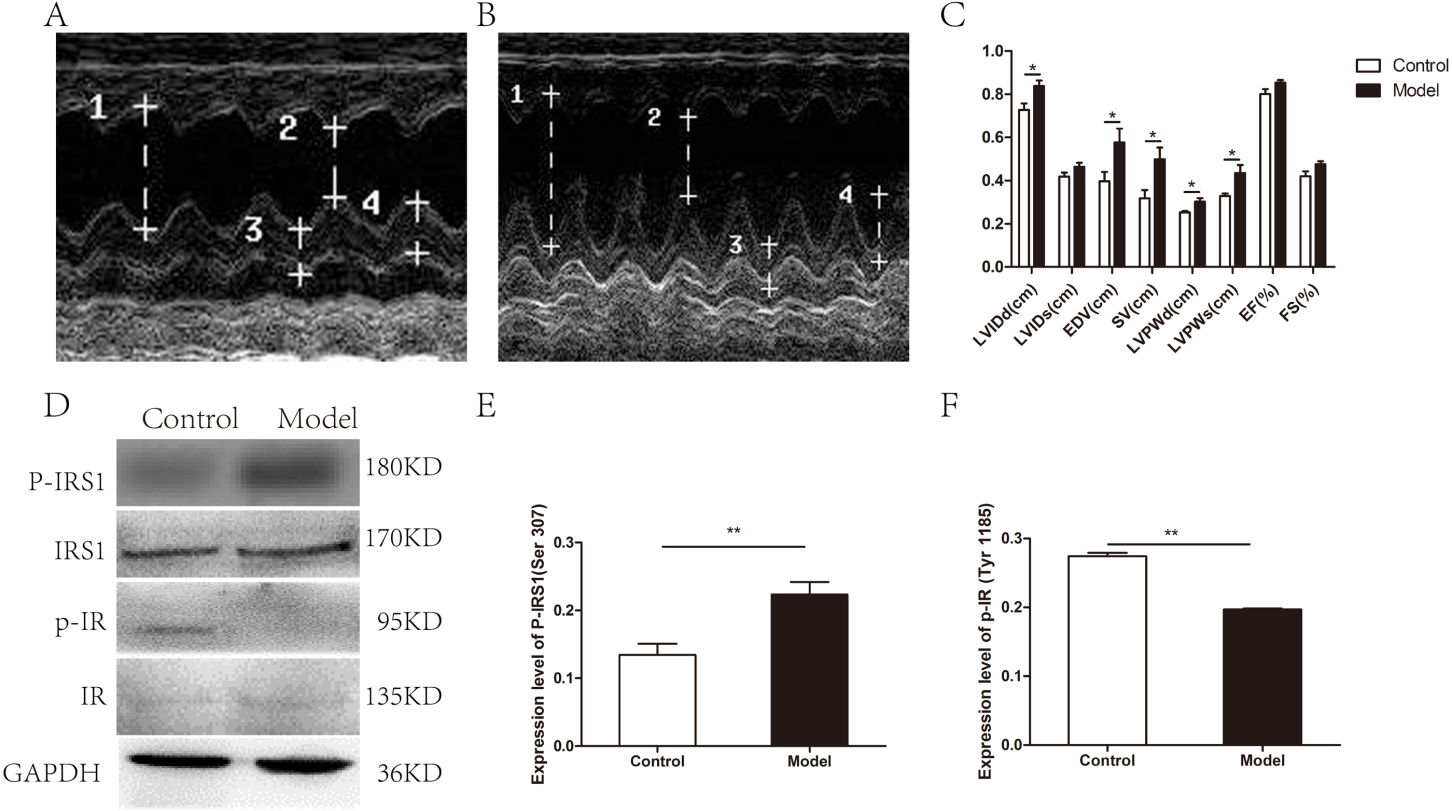
Cardiac structure and function and the myocardial insulin sensitivity were attenuated in chronic alcohol intake rats. (A) Echocardiogram in a control animal. (B) Echocardiogram in a rat that has consumed alcohol for 20 weeks. 1 left ventricular internal diastolic diameter (LVIDd), two left ventricular end systolic diameter (LVIDs), three left ventricular posterior wall depth (LVPWd), 4 left ventricular posterior wall thickness at end-systole (LVPWs). (C) Variation of cardiac function indexes after a prolonged drinking period (*n* = 6 per group); data are presented as mean ± SD. (D) Immunoblot of p-IRS1(ser 307) and p-IR (Tyr 1185) after treatment of rats with ethanol for 20 weeks. (E) The band intensities of p-IRS1(ser 307) normalized to IRS1. (F) The band intensities of p-IR (Tyr 1185) normalized to IR (*n* = 6 per group). Data are shown as mean ± SD. ^∗^*p* < 0.05; ^∗∗^*p* < 0.01.

As determined by UCG, 20 weeks alcohol intakes resulted in a compensated state of volume-overload heart failure as shown by the absence of overt systolic dysfunction (Figs. 1A–1C). A significant difference was documented between the model group and the control group for the following parameters: LVIDd (0.84 ± 0.06 cm vs. 0.73 ± 0.07 cm, respectively; *p* = 0.019), LVPWd (0.30 ± 0.03 cm vs. 0.25 ± 0.02 cm, respectively; *p* = 0.02), LVPWs (0.44 ± 0.09 cm vs. 0.33 ± 0.03 cm, respectively; *p* = 0.033), EDV(0.58 ± 0.16 ml vs. 0.40 ± 0.11 ml, respectively; *p* = 0.043), SV(0.50 ± 0.13 ml vs. 0.32 ± 0.09 ml, respectively; *p* = 0.022). However, no significant changes were observed in LVIDs (0.46 ± 0.05 cm vs. 0.42 ± 0.05 cm, respectively; *p* = 0.126), EF (0.85 ± 0.03 vs. 0.80 ± 0.06, respectively; *p* = 0.070) and FS (0.48 ± 0.04 vs. 0.42 ± 0.05, respectively; *p* = 0.069). In addition, body weight and heart weight were measured before and up to 20 weeks alcohol consumption. Heart weights normalized to body weight (BW) showed the same tendency (*p* = 0.011), which indicated cardiac hypertrophy with chronic alcohol consumption (Table S1).

**Table 1:** The primer sequences.

Gene Name	Forward primer	Reverse primer
*rheb*	GCTGATCACAGTAAATGGGCAAGA	CCAACAGCTTGCCGTGGATA GACTT
*rictor*	CCCCACTCAAAGCACACATC	TCCCAGCCCACACTC
*S6k2*	CTACTGAGTGTTCTGGGCAAGG	TGGCACTGCATACAATCTTGG
*GAPDH*	CAAGTTCAACGGCACAGTCAAG	ACATACTCAGCACCAGCATCAC
*U6*	CAGCACATATACTAAAATTGGAAGG	ACGAATTTGCGTGTCATCC
*miR-155-5p*	CGGCGGTTAATGCTAATTGTGAT	GTGCAGGGTCCGAGGT

**Table 2:** Elementary procedure of Real-Time PCR.

Stage	Temperature	Reaction time
Initial denaturation	95 °C	5min
circulation (44 cycles)	95 °C	15s→56 °C 30s→72 °C 20s
melting	75 °C→95 °C	Rise by 1 °C every 20s

Next, the expression levels of phosphorylated insulin receptor substrate 1 (p-IRS1) and phosphorylated insulin receptor (p-IR) were determined by western blotting to evaluate myocardial insulin sensitivity. The expression of p-IRS1(ser 307) in the model group was remarkedly increased than that in the control group with the total IRS1 amount unchanged, whereas p-IR (Try 1185) level was remarkedly decreased with the total IR amount unchanged (Figs. 1D–1F).

To evaluate the effect of chronic alcohol drinking on insulin sensitivity, OGTT test was carried out, AUC and HOMA-IR were calculated. Compared with the control group, long-term drinking lead to lower insulin sensitivity in model rat. Rats in the chronic alcohol consumption groups demonstrated elevated fasting blood glucose (FBG), and increased HOMA-IR compared to the control group (Table S1). Rats in the chronic alcohol consumption groups demonstrated elevated fasting blood glucose, and increased HOMA-IR compared to the control group (Table S1).

### mTOR signaling pathway was activated in cardiac tissues in chronic alcohol intake rats

**Figure 2:**
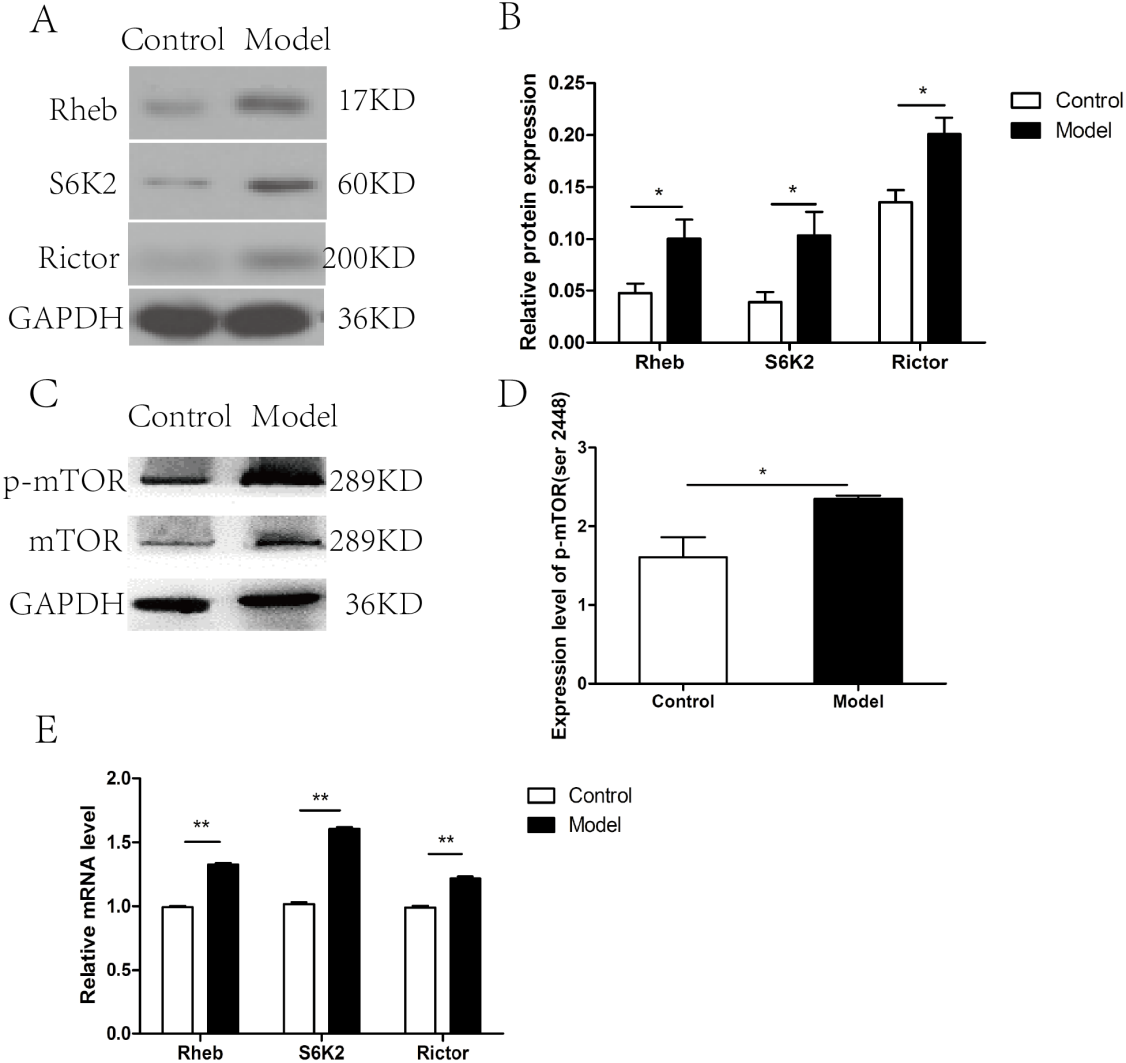
mTOR signaling pathway was activated in cardiac tissues in chronic alcohol intake rats. (A) Western blotting images of Rheb, S6K2 and Rictor in heart tissue samples from model rats after 20 weeks ethanol consumption and the control rats. (B) Relative band intensities of Rheb, S6K2 and Rictor, in heart tissue samples. (C) Western blotting images of p-mTOR (ser 2448) in heart tissue samples. (D) Relative band intensities of p-mTOR (ser 2448). (E) Real-time quantitative PCR for Rheb, S6K2, and Rictor in heart tissue samples (*n* = 6 per group). Data are presented as mean ± SD. ^∗^*p* < 0.05; ^∗∗^*p* < 0.01.

The expression of the members of mTOR complex, Rictor, Rheb, and S6K2 were investigated at both mRNA and protein level. The expression level of Rictor, Rheb, and S6K2 proteins were elevated after chronic ethanol exposure relative to that in control rats (*p* < 0.05) (Figs. 2A–2B), which was correspond to the increase in the p-mTOR (ser 2448) (Figs. 2C–2D). Furthermore, RT-PCR revealed that the mRNA levels of Rictor, Rheb, and S6K2 were dramatically increased in model group than that in the control group (Fig. 2E).

### miR-155-5p was up-regulated in chronic alcohol intake rats

**Figure 3:**
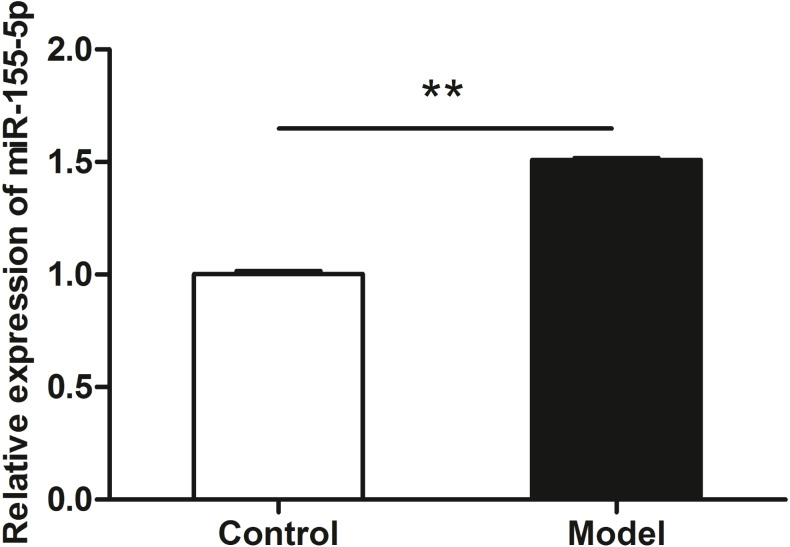
miR-155-5p was upregulated in chronic alcohol intake rats. RT-PCR analysis showed about 1.5-fold in the heart in alcohol treated rat compared with the control group. Data were expressed as mean ± SD (*n* = 6 per group). ^∗∗^*p* < 0.01.

qRT-PCR data revealed the upregulation of miR-155-5p in heart tissue in chronic alcohol intake rats. As shown in Fig. 3, after 20 weeks of ethanol consumption, the expression of miR-155-5p in cardiac tissues was elevated up to 150% in the model group compared to that in the control group (*p* < 0.001).

### The myocardial insulin sensitivity was ameliorated after miR-155-5p upregulation

To clarify the role of miR-155-5p in this process and to identify potential therapeutic targets for ACM, Wistar rats treated with alcohol were infected with an AAV-miR-155-5p or an AAV-NC. qRT-PCR confirmed the significant overexpression of miR-155-5p. As shown in Fig.S1, miRNA-155-5p level was remarkably upregulated by 2.67-fold in the heart of the AAV-miR-155 group relative to the AAV-NC group.

**Figure 4:**
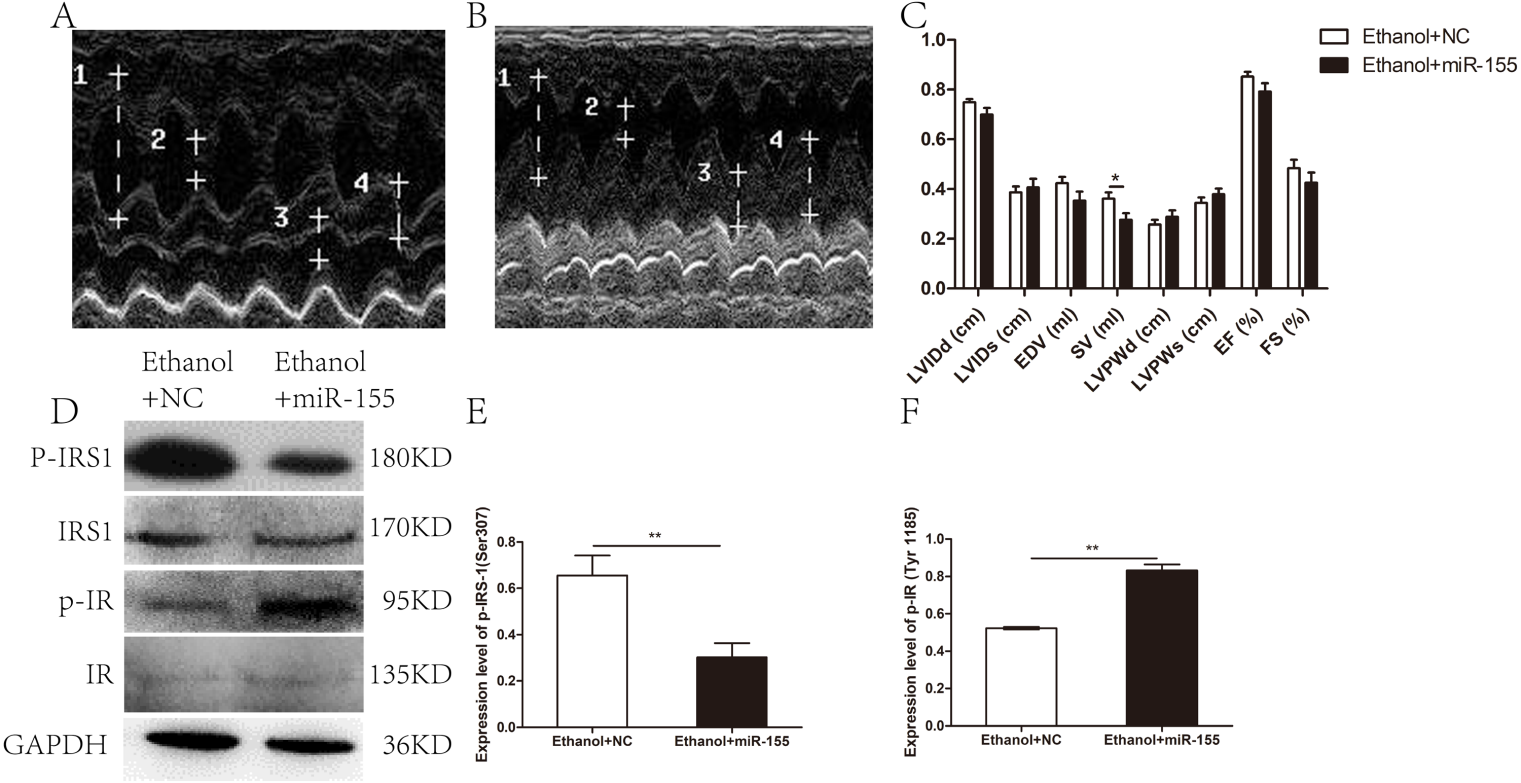
Effects of miR-155-5p upregulation on cardiac function and myocardial insulin sensitivity. (A) Echocardiogram in the AAV-NC animal. (B) Echocardiogram in the AAV-miR-155-5p group. one left ventricular internal diastolic diameter (LVIDd), two left ventricular end systolic diameter (LVIDs), three left ventricular posterior wall depth (LVPWd), 4 left ventricular posterior wall thickness at end-systole (LVPWs). (C) Variation of cardiac function indexes after a prolonged drinking period (*n* = 8 per group). (D) p-IRS1(ser 307) and p-IR (Tyr 1185) levels following an AAV-miR-155 delivery were assessed by western blotting (*n* = 8 per group). (E) Relative band intensities of p-IRS1(ser 307) normalized to IRS1. (F) The band intensities of p-IR (Tyr 1185) normalized to IR (*n* = 8 per group). Data are presented as mean ± SD. ^∗^*p* < 0.05; ^∗∗^*p* < 0.01.

Cardiac geometry and function were evaluated to identify the potential benefit of AAV-miR-155-5p transfection. As shown in Figs. 4A–4C, a significant difference was documented between the AAV-miR-155 group and AAV-NC group for SV (0.28 ± 0.08 ml vs. 0.36 ± 0.07 ml, respectively; *p* = 0.037). However, no significant difference was documented between the AAV-miR-155-5p group and AAV-miR-NC group for the following parameters: LVIDd (0.70 ± 0.07 cm vs. 0.75 ± 0.04 cm, respectively; *p* = 0.117), LVIDs (0.41 ± 0.10 cm vs. 0.39 ± 0.07 cm, respectively; *p* = 0.647), LVPWd (0.29 ± 0.08 cm vs. 0.26 ± 0.05 cm, respectively; *p* = 0.383), LVPWs (0.38 ± 0.07 vs. 0.35 ± 0.06, respectively; *p* = 0.305), EDV(0.35 ± 0.11 ml vs. 0.42 ± 0.07 ml, respectively; *p* = 0.134), EF (0.79 ± 0.09 vs. 0.85 ± 0.05, respectively; *p* = 0.143) and FS (0.43 ± 0.12 vs. 0.48 ± 0.10, respectively; *p* = 0.288).

The levels of p-IRS1 (ser 307) were remarkedly decreased in AAV-miR-155-5p group than that in the AAV-NC group, whereas the levels of p-IR (Tyr 1185) were remarkedly increased (Figs. 4D–4F). However, miR-155-5p upregulation did not ameliorate glucose tolerance or insulin sensitivity (Table S1).

### The mTOR signaling pathway was negatively regulated by over-expressed miR-155-5p

**Figure 5:**
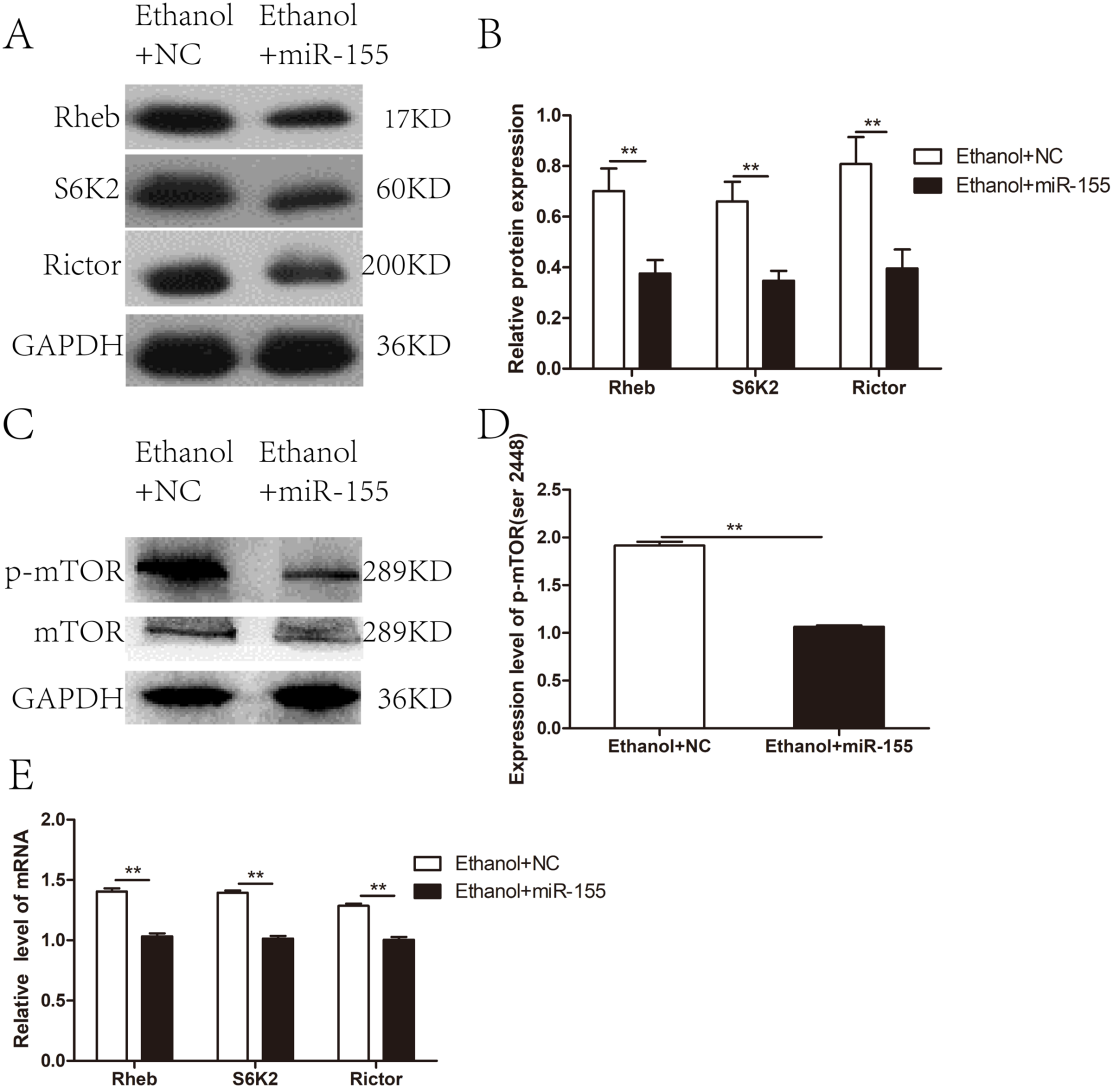
Effects of miR-155-5p upregulation on the mTOR signaling pathway. (A) The expression of Rheb, S6K2 and Rictor in rats heart tissue at 15days post-injection was detected by western blot (*n* = 8 per group). (B) Relative band intensity of Rheb, S6K2 and Rictor in ACM heart tissue (*n* = 8 per group). (C) Western blot of p-mTOR (ser 2448) in ACM heart tissue (*n* = 8 per group) transfected with AAV-NC and AAV-miR-155 (*n* = 8 per group). (D) Relative band intensity of p-mTOR (ser 2448) normalized to mTOR. (E) Real-time quantitative PCR for Rheb, S6K2, and Rictor in heart tissue samples (*n* = 8 per group). Data are presented as mean ± SD. ^∗^*p* < 0.05; ^∗∗^*p* < 0.01.

To investigate whether miR-155-5p overexpression improves myocardial insulin sensitivity in alcohol drinking rats via downregulation of the mTOR pathway, we assessed the expression of molecules related to mTOR signaling in AAV-miR-155-5p infected heart tissue samples. Western blotting showed that the levels of Rictor, Rheb, and S6K2 proteins were significantly downregulated in miR-155-5p overexpressing heart tissue (Figs. 5A–5B). Meanwhile, p-mTOR (ser 2448) was significantly downregulated in miR-155-5p overexpressing heart tissue samples (Figs. 5C–5D). RT-PCR analysis confirmed that the mRNA levels of Rictor, Rheb, and S6K2 were significantly downregulated in miR-155-5p overexpressing heart tissue (Fig. 5E).

## Discussion

We aimed to explore the role of miR-155-5p up-regulation in chronic alcohol consumption induced myocardial insulin resistance and the mechanisms of mTOR signaling pathway involved in this process. Our data revealed the following aspects. Firstly, chronic alcohol consumption lead to myocardial insulin resistance. Secondly, the mTOR signaling pathway was activated in vivo, along with miR-155-5p up-regulation. Thirdly, miR-155-5p overexpression protect against myocardial insulin resistance, accompanied by negatively regulated mTOR signaling pathway in alcohol-drinking rats. It is conceivable based on our findings that miR-155-5p upregulation ameliorated ethanol induced myocardial insulin resistance via the mTOR signaling pathway. But the causal relationship between miR-155 and myocardial insulin resistance remains unknown. Further investigations are needed to precisely clarify the role of miR-155 in ACM and the underlying mechanisms.

Chronic ethanol intake can cause cardiac insulin sensitivity defect and systolic dysfunction, and the overexpression of aldehyde dehydrogenase gene 2 could antagonize this effect of alcohol by improving insulin signaling at the levels of IR, IRS, Akt, FOXO3a and JNK (Li et al., 2009). Studies have shown that heavy drinking reduces left ventricular ejection fraction, and higher alcohol consumption increases the risk of cardiac systolic dysfunction (Van Oort et al., 2020). In our study, model rats exhibited significantly subclinical left-ventricle (LV) systolic dysfunction, with an increase in LVIDd, EDV, and SV. However, no significant changes were observed in LVIDs, EF and FS. In addition, our results confirm that long-term ethanol consumption induces insulin resistance and thus diabetes pathogenesis risk, which was consistent with Yang, Wu & Leung (2020). In view of the heart dysfunction and myocardial insulin resistance caused by ethanol, reducing alcohol consumption might be metabolically favorable for human future glucose metabolism.

miR-155 is an immunomodulatory miRNA whose function has been extensively investigated in numerous pathophysiological processes such as stroke, atherosclerosis, and in conditions associated with inflammatory response. It seems that miR-155 always be an “evildoer”. miR-155 is highly expressed in alcohol-exposed RAW 264.7 macrophages, isolated hepatocytes and Kupffer cells of alcohol fed mice (Bala et al., 2011; Bala & Szabo, 2012); and its high level in macrophages magnifies the production of tumor necrosis factor alpha (TNF-*α*) (Bala et al., 2011), which is the important mediator of hepatic steatosis, inflammation and liver cell death in alcoholic liver disease. However, in murine non-alcoholic fatty liver model induced by high-fat diet, miR-155 overexpression can alleviate it via lipid metabolism (Lin et al., 2015). miR-155 seems to be an be an “evildoer”, and its function seems to be diverse in different etiology and disease context (Hartmann & Tacke, 2016). Previous research indicated miR-155 deficiency leads to the increased white adipocyte hypertrophy, obesity and hyperinsulinemia (Virtue et al., 2017). In addition, Lin et al. (2016) demonstrated that entire body transgenic overexpression of miR-155 led to hypoglycemia, improved glucose tolerance and insulin sensitivity, enhanced glycolysis, and insulin-induced AKT and IRS-1 phosphorylation in the liver, adipose tissue, and skeletal muscle. Another study (Zhang et al., 2016) reported that in adipocytes, the miR-155 generated by adipocyte-derived microvesicles can mediated M1 macrophage polarization, and inhibit insulin signaling in adipocytes. This reveals a novel mechanism that obesity induces an imbalance in the M1-to-M2 macrophage ratio in adipose tissue, resulting in chronic inflammation and local insulin resistance. Oxidative stress is involved in several processes including cancer, aging and cardiovascular disease. H_2_O_2_ stimulation increased the expression of miR-155 in cardiomyocyte progenitor cells (CMPCs), and its overexpression attenuated necrotic cell death via targeting receptor interacting protein 1 (RIP1), which provides a theoretical basis for the protective effect of miR-155 on the heart (Liu et al., 2012; Liu et al., 2011). However, the function of miR-155 in regulating myocardial insulin sensitivity and the underlying mechanism remains unknown.

Adeno-associated virus is a promising vector for gene therapy, and AAV-mediated gene transfer long-term modulates of gene expression for its durable protein expression in targeted tissues. Previous studies have showed that AAV9 injection was an effective tool for gene delivery in the heart in vivo (Wang, Guo & Pu, 2021), and suggested the effect of adeno-associated virus (AAV9) system injection on the cardiac function. Li et al. (2020a) reported that administration of Zinc finger protein ZBTB20 by AAV9 system alleviate cardiac remodeling post myocardial infarction and thus can be considered as a promising treatment strategy for heart failure. Wei et al. (2020) reported overexpression of secreted frizzled-related protein 2 by AAV9 tail vein injection alleviated cardiomyocyte hypertrophy and interstitial fibrosis. In the present study, adeno associated virus (AAV-miR-155) myocardial injection upregulated miR-155-5p level, alleviated the increase of SV, improved myocardium insulin sensitivity, and intriguingly without improvement of glucose tolerance and HOMA-IR. Our data suggested chronic alcohol consumption not only impaired systemic insulin sensitivity and myocardial local insulin sensitivity, but also attenuated cardiac function in alcohol intake rats. 20 weeks alcohol resulted in a compensated state of volume-overload heart failure, as shown by an increase of LVDd, EDV, SV, LVPWd and LVPWs. miR-155-5p upregulation contributed to an alleviated increased stroke volume and myocardial insulin sensitivity, however without an ameliorated systemic insulin sensitivity (Table S1). Taken together, these data demonstrated that miR-155 is a positive regulator of myocardial insulin sensitivity with potential applications in ACM treatment.

miR-155 expression varies in different models. miR-155 was remarkably downregulated in the left ventricular specimens obtained from streptozotocin-induced diabetic mice (Costantino et al., 2016). However, Huang, Hao & Hu (2019) demonstrated that miR-155 was highly expressed in ischemia/reperfusion mice; furthermore, the upregulation of miR-155 impaired cardiac function and increased cardiomyocyte apoptosis by negatively regulating silent information regulator 1(SIRT1). In patients with coronary heart disease (CHD), miR-155 was higher expressed and that serum miR-155 levels might serve as a novel biomarker for the evaluation of CHD severity (Qiu & Ma, 2018). In our study, miR-155-5p levels were significantly higher expressed in the heart of chronic alcohol intake rats; interestingly, miR-155-5p overexpression ameliorated myocardial insulin resistance, suggesting that miR-155-5p upregulation in chronic alcohol intake rats represented a stress response to ethanol exposure. However, no significant changes in cardiac function was observed after transfection miR-155-5p indeed, possibly due to the brief duration of virus action. The ameliorated myocardial insulin sensitivity by miR-155 over-expression is a sensitive index for the improvement of cardiac insulin signaling. It may take more than 14 days for myocardial remodeling and improvement of cardiac function. The ameliorated myocardial insulin sensitivity by miR-155 over-expression is a sensitive index for the improvement of cardiac function. It is necessary to carry out experiments in gene model animal in further.

As a central regulator of cell growth, mTOR plays a significant role in insulin target tissues such as muscle and liver. Inhibition of the mTOR pathway using rapamycin prevented insulin resistance caused by chronic hyperinsulinemia in liver and muscle (Ueno et al., 2005). In our previously study (Li et al., 2020b), miR-155-5p upregulation increased the glucose uptake of H9C2 cells, suppressed the mTOR signaling pathway and miR-155-5p downregulation decreased the glucose uptake of H9C2 cells, activated the mTOR signaling pathway, which suggested miR-155 regulated insulin signaling pathway via mTOR signaling. In the present study, alcohol intakes upregulated the miR-155-5p expression level, and the upregulation of miR155 should be protective to inactivate the mTOR signaling and ameliorate myocardial insulin resistance. We found that miR-155-5p was upregulated by mTOR and to regulate in turn S6K2, Rheb and Raptor, which they showed to be direct targets of miR-155-5p. Modulation of mTOR and miR-155-5p in vitro demonstrated the existence of a feedback crosstalk between mTOR pathway and insulin pathway. Thus, the in vivo administration of miR-155-5p after subjecting the rats to alcohol injury, ameliorated chronic alcohol consumption-induced myocardial insulin resistance. However, miR-155-5p was found dramatically upregulated in the hearts of alcohol intakes rats’ model, and it seemed that endogenous levels of miR-155-5p were lower than the levels reached after exogenous miR-155-5p administration, accounting for its failure to protect from the heart function. So miR-155 endogenous upregulation in a physiological range is not enough to reverse the damage caused by ethanol and the specific mechanism still needs to be further explored. Based on these findings, we conclude that miR-155-5p upregulation ameliorates chronic alcohol consumption-induced myocardial insulin resistance via the mTOR signaling pathway, which may provide a potential therapeutic target for ACM.

One of the strengths of this study is its novelty. To the best of our knowledge, this study presents the first evidence that miR-155 regulates insulin signaling in the heart tissue of alcohol-drinking rats, thereby further elucidating the role of miR-155 in the regulation of mTOR signaling and ACM pathogenesis. However, this study also has some limitations. First, the mechanism through which miR-155 regulates the insulin sensitivity of myocardial cells, needs to be investigated in depth. Second, it would be useful to apply the isotope labeled cardiac perfusion approach to elucidate the role of miR-155 in the regulation of insulin responsiveness in myocardial cells. However, considering the experimental platform constraints and the radioactive contamination, we did not adopt this approach. Thirdly, more time points should be set to evaluate the effect of viral transfection on alcoholic cardiomyopathy. Despite such limitations, our study clearly showed that miR-155-5p upregulation ameliorated myocardial insulin resistance via the mTOR signaling pathway. And, additional samples from clinical patients will be an area of our future investigation.

## Conclusions

Overall, our data demonstrated that miR-155-5p upregulation may protect against myocardial insulin resistance in chronic alcohol intake rats via the mTOR signaling pathway, which might provide a potential therapeutic target for ACM.

##  Supplemental Information

10.7717/peerj.10920/supp-1 Figure S1 Successful overexpression of miR-155-5p by adeno-associated virus pre-miR-155-5p treated RT-PCR analysis showed a 2.67-fold change of miR-155-5p level compared with the AAV-NC group. Data were expressed as mean ± SD (*n* = 8 per group). ^∗∗^*p* < 0.01.

10.7717/peerj.10920/supp-2 Table S1 Summary information about rat’s heart weight, systemic glucose tolerance and insulin sensitivity

10.7717/peerj.10920/supp-3 Supplemental Information 3 Checklist

10.7717/peerj.10920/supp-4 Supplemental Information 4 Raw data

10.7717/peerj.10920/supp-5 Supplemental Information 5 B-ultrasound pictures and Western blot

## Additional Information and Declarations

### Data Availability

The following information was supplied regarding data availability: The raw measurements are available in the Supplemental Files.
