# Supplementary material for: miR-155-5p upregulation ameliorates myocardial insulin resistance via mTOR signaling in chronic alcohol drinking rats
Source: PeerJ. 2021 Apr 5;9:e10920. doi: 10.7717/peerj.10920 (PMC8029671; doi:10.7717/peerj.10920)
Supplement: Table S1 [file peerj-09-10920-s002.docx]

**Supplementary table 1** Summary information about rat’s heart weight, systemic glucose tolerance and insulin sensitivity

| **groups** | **Body weight(g)** | | | **heart-weight(mg)** | **Heart weight index**  **(mg/g)** | **FBG**  **(mmol/L)** | **AUC** | **HOMA-IR** |
| --- | --- | --- | --- | --- | --- | --- | --- | --- |
|  | **initial** | **20-week** | **final** |  |  |  |  |  |
| **Control** | 247±16.06 | 428±21.70**^*^** | 437±28.05**^*^** | 857±35.09 | 1.97±0.16 | 5.70±0.40^**^ | 16.13±2.33 | 1.88±0.24^*^ |
| **Model** | 250±15.86 | 392±20.06 | 402±27.38 | 867±35.49 | 2.16±0.18 | 6.65±0.55 | 15.96±3.03 | 2.96±1.14 |
| **AAV-miR-scramble** | 249±16.32 | 396±23.00 | 391±23.80 | 864±30.56 | 2.21±0.19 | 4.93±0.58**^#^** | 15.29±2.31 | 3.04±1.10 |
| **AAV-miR-155-5p** | 249±12.69 | 398±21.76 | 392±21.41 | 859±32.20 | 2.18±0.18 | 5.74±0.58 | 16.18±1.63 | 2.31±0.52 |

The values are mean ± SD. ******p*<0.05, *******p*<0.01 Control vs Model; **^#^***p*<0.05 AAV-miR-scramble vs AAV-miR-155-5p.
