# Supplementary figures and images for: miR-155-5p upregulation ameliorates myocardial insulin resistance via mTOR signaling in chronic alcohol drinking rats
Source: PeerJ. 2021 Apr 5;9:e10920. doi: 10.7717/peerj.10920 (PMC8029671; doi:10.7717/peerj.10920)

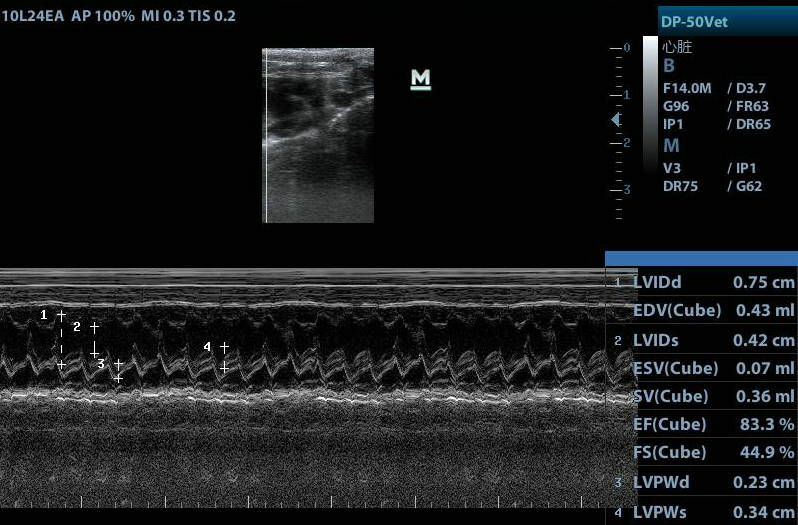

Supplement: Supplemental Information 5 [file peerj-09-10920-s005.zip › data/B │1⁄4/AAV NC/B-1.JPG]

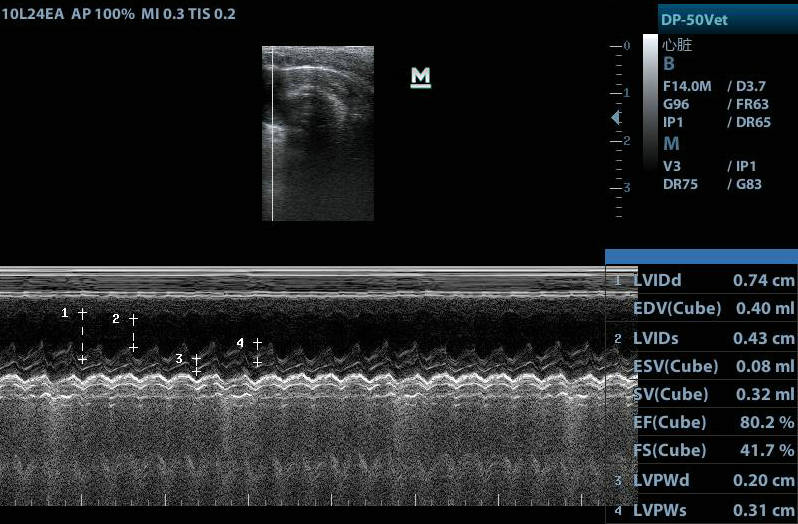

Supplement: Supplemental Information 5 [file peerj-09-10920-s005.zip › data/B │1⁄4/AAV NC/B-2.JPG]

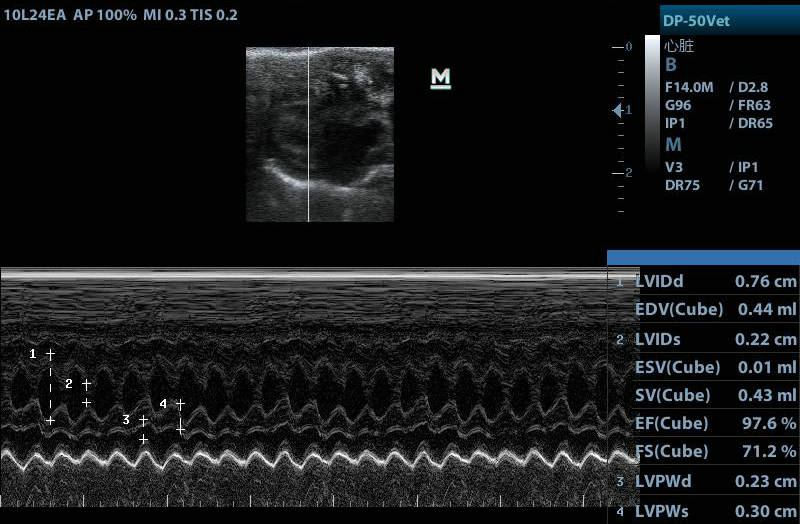

Supplement: Supplemental Information 5 [file peerj-09-10920-s005.zip › data/B │1⁄4/AAV NC/B-3.JPG]

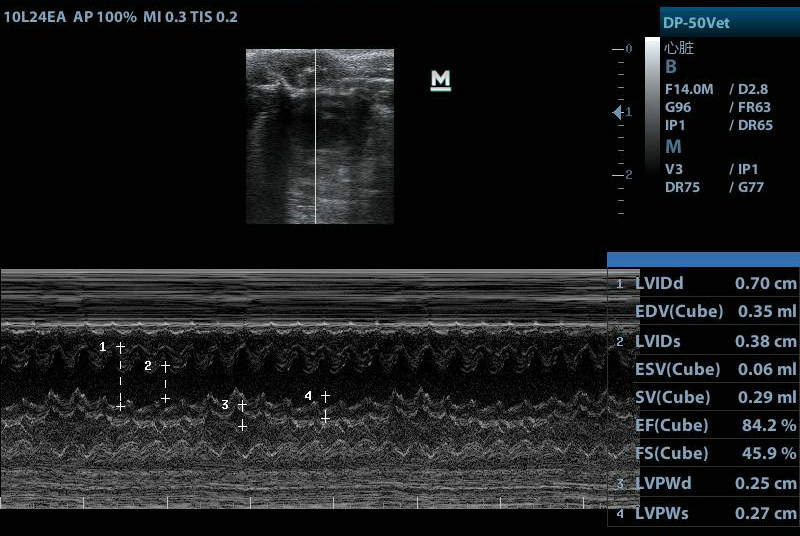

Supplement: Supplemental Information 5 [file peerj-09-10920-s005.zip › data/B │1⁄4/AAV NC/B-4.JPG]

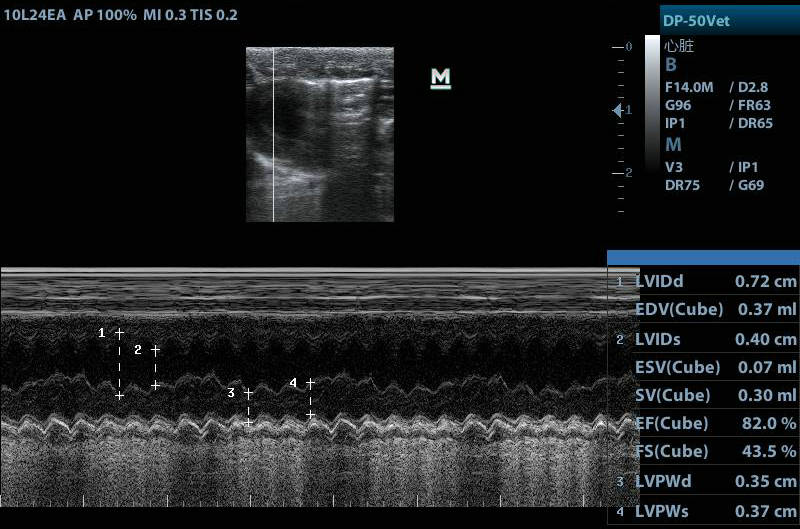

Supplement: Supplemental Information 5 [file peerj-09-10920-s005.zip › data/B │1⁄4/AAV NC/B-5.JPG]

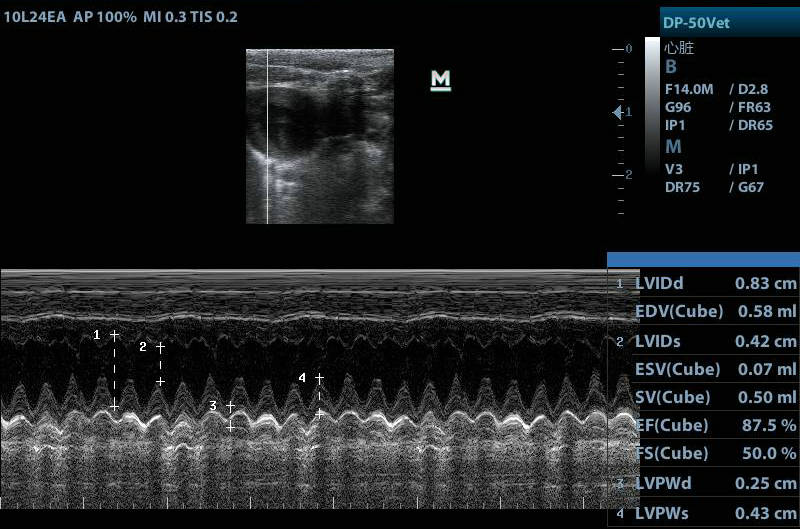

Supplement: Supplemental Information 5 [file peerj-09-10920-s005.zip › data/B │1⁄4/AAV NC/B-6.JPG]

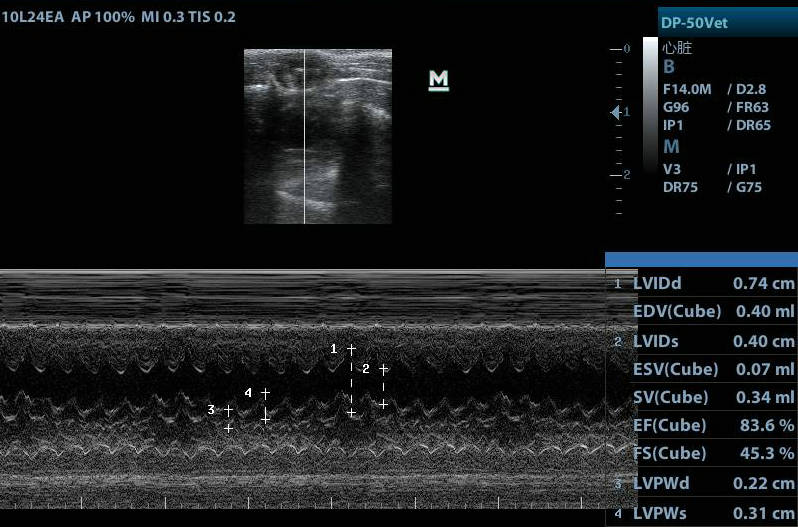

Supplement: Supplemental Information 5 [file peerj-09-10920-s005.zip › data/B │1⁄4/AAV NC/B-7.JPG]

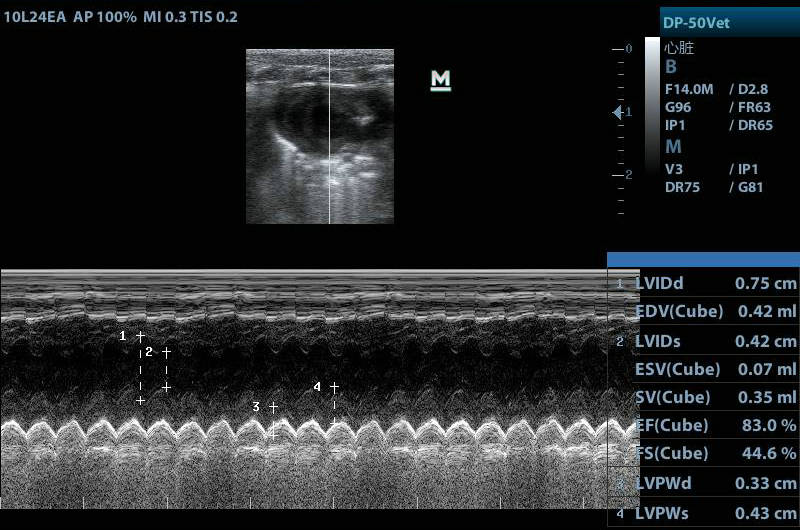

Supplement: Supplemental Information 5 [file peerj-09-10920-s005.zip › data/B │1⁄4/AAV NC/B-8.JPG]

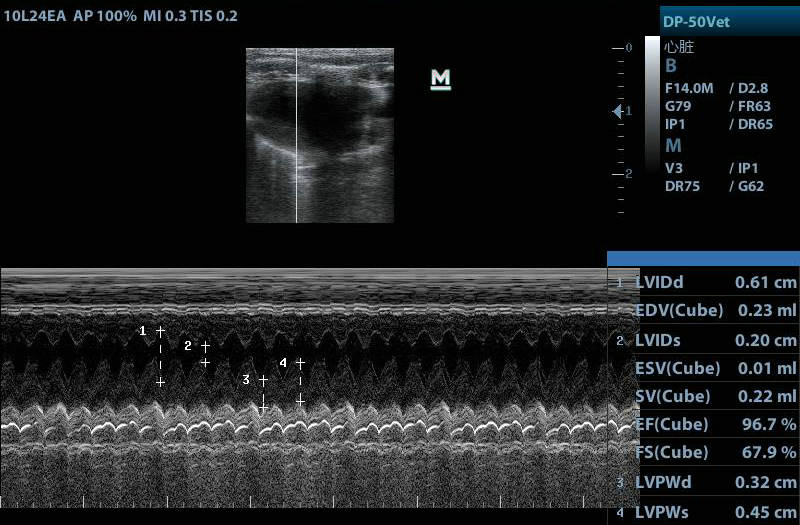

Supplement: Supplemental Information 5 [file peerj-09-10920-s005.zip › data/B │1⁄4/AAV/A-1.JPG]

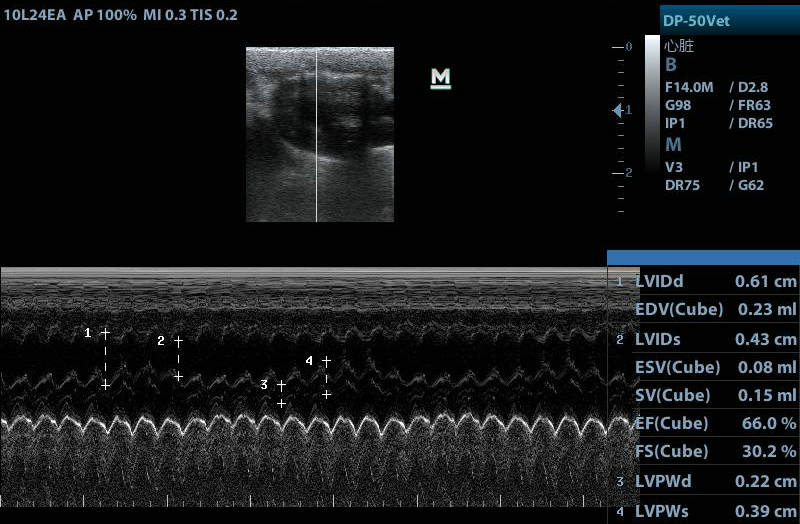

Supplement: Supplemental Information 5 [file peerj-09-10920-s005.zip › data/B │1⁄4/AAV/A-2.JPG]

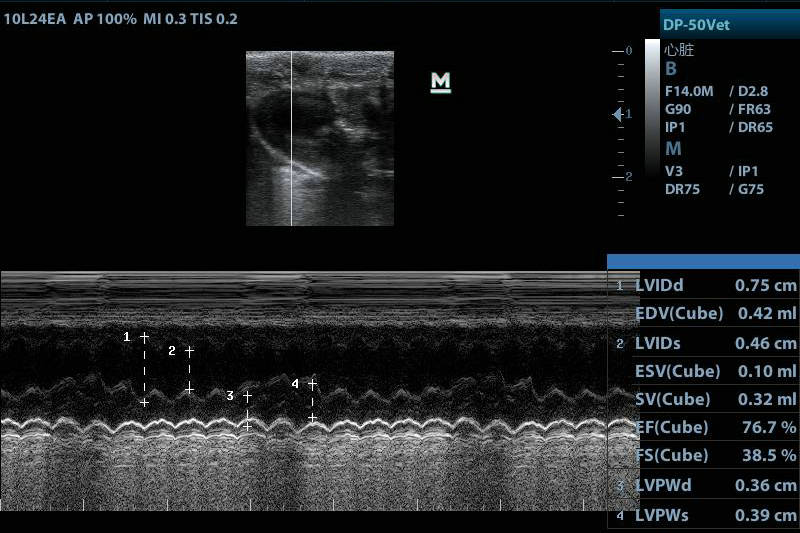

Supplement: Supplemental Information 5 [file peerj-09-10920-s005.zip › data/B │1⁄4/AAV/A-3.JPG]

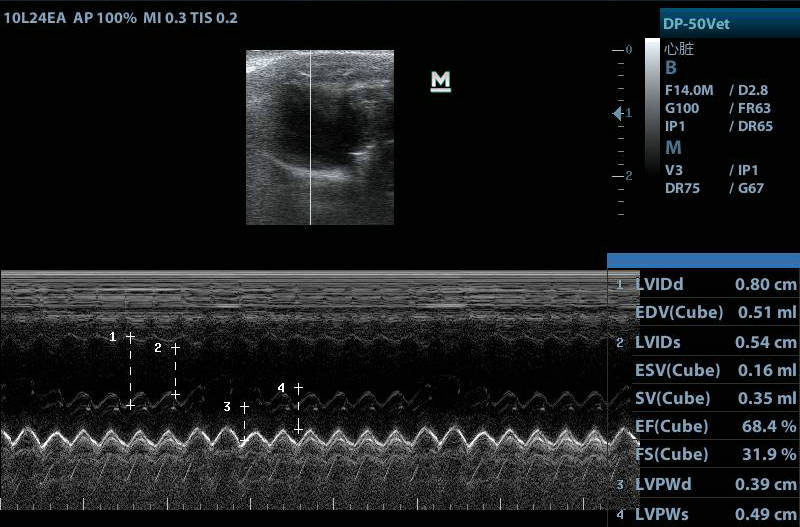

Supplement: Supplemental Information 5 [file peerj-09-10920-s005.zip › data/B │1⁄4/AAV/A-4.JPG]

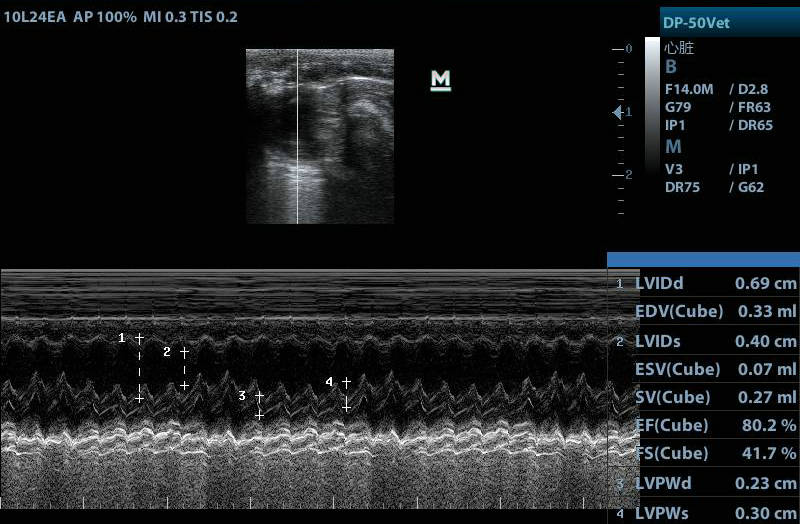

Supplement: Supplemental Information 5 [file peerj-09-10920-s005.zip › data/B │1⁄4/AAV/A-5.JPG]

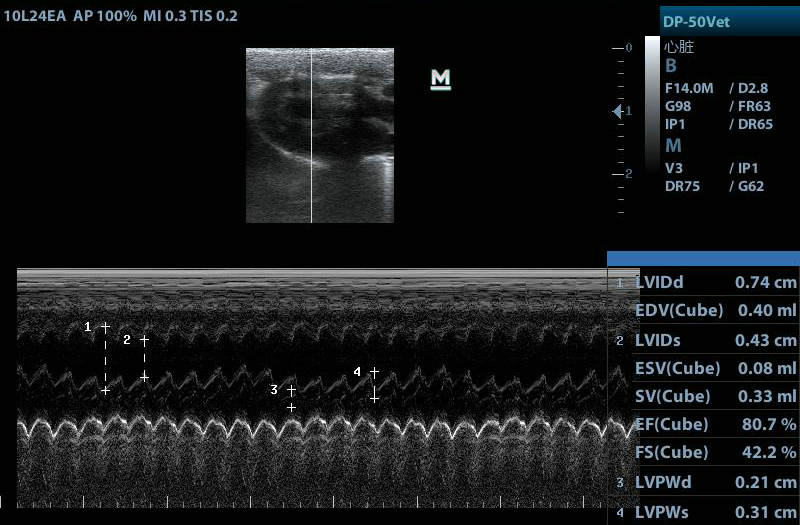

Supplement: Supplemental Information 5 [file peerj-09-10920-s005.zip › data/B │1⁄4/AAV/a-6.JPG]

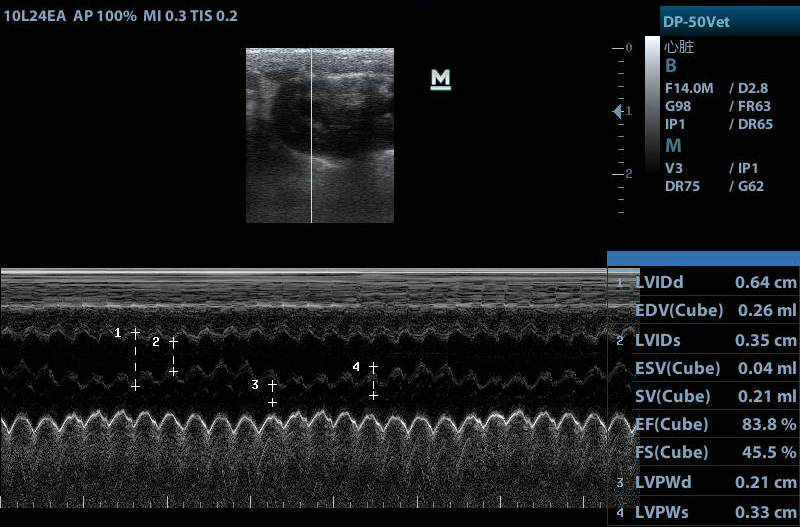

Supplement: Supplemental Information 5 [file peerj-09-10920-s005.zip › data/B │1⁄4/AAV/a-7.JPG]

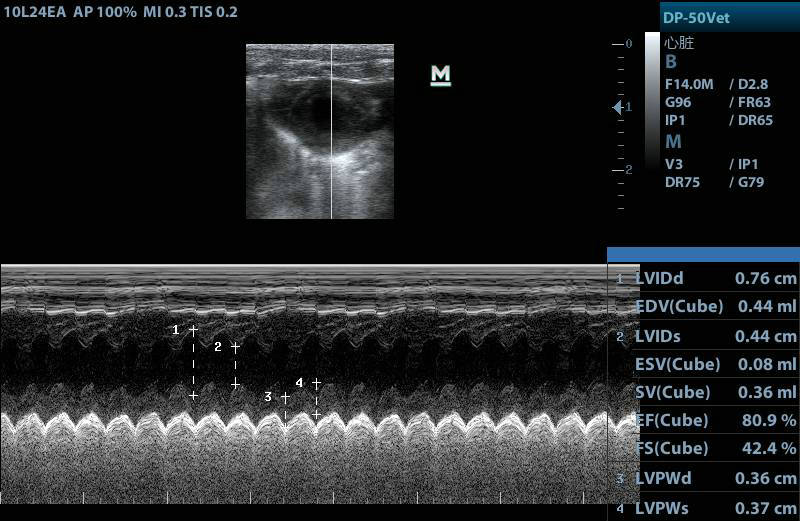

Supplement: Supplemental Information 5 [file peerj-09-10920-s005.zip › data/B │1⁄4/AAV/A8.JPG]

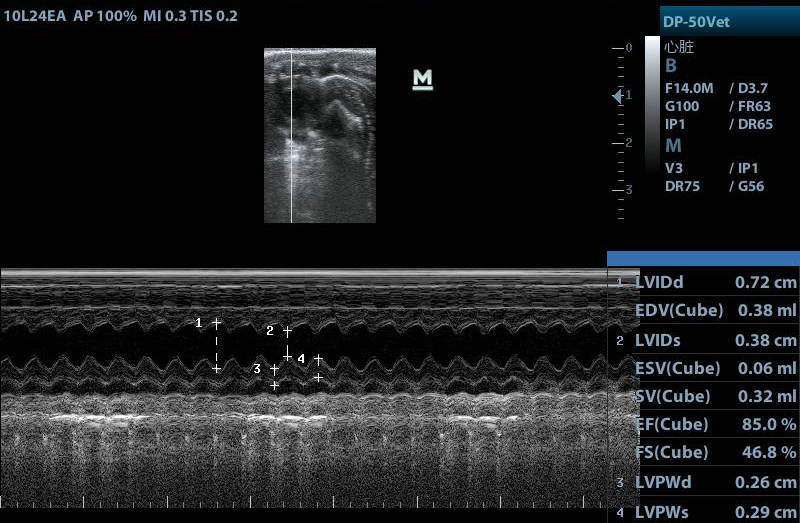

Supplement: Supplemental Information 5 [file peerj-09-10920-s005.zip › data/B │1⁄4/control/A1.JPG]

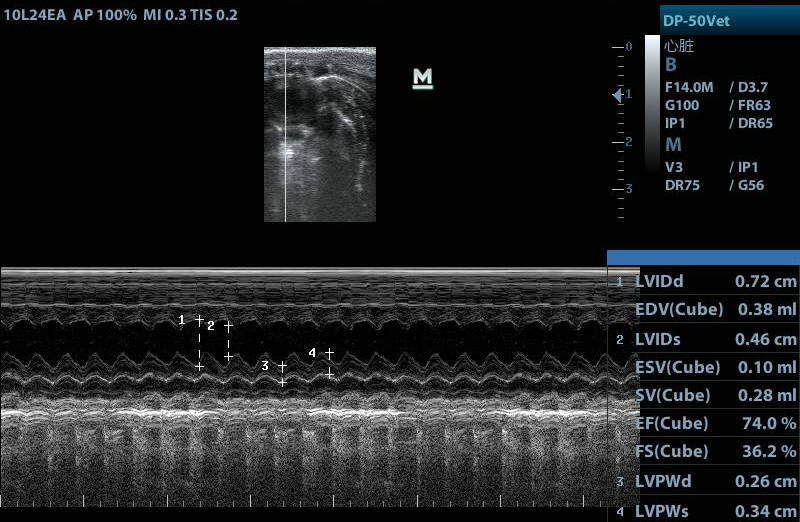

Supplement: Supplemental Information 5 [file peerj-09-10920-s005.zip › data/B │1⁄4/control/A2.JPG]

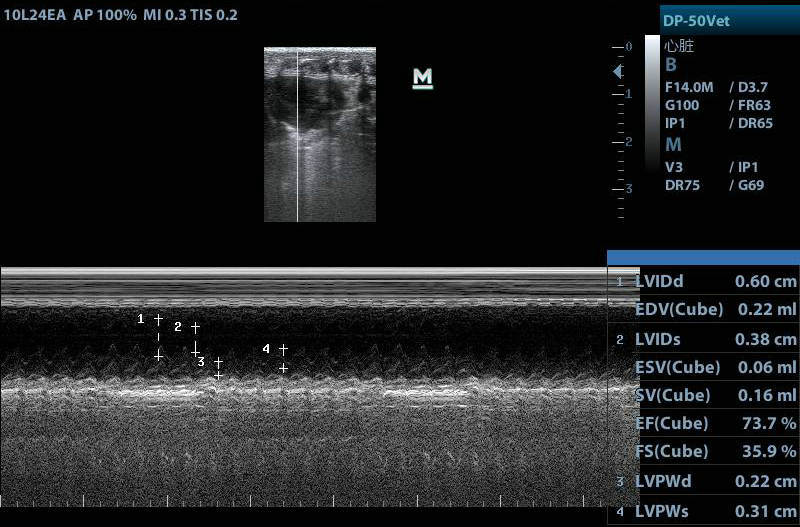

Supplement: Supplemental Information 5 [file peerj-09-10920-s005.zip › data/B │1⁄4/control/A3.JPG]

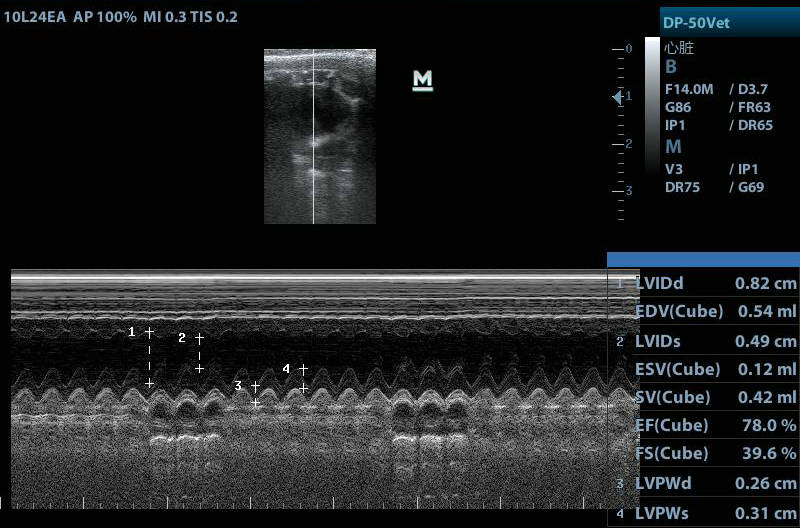

Supplement: Supplemental Information 5 [file peerj-09-10920-s005.zip › data/B │1⁄4/control/A4.JPG]

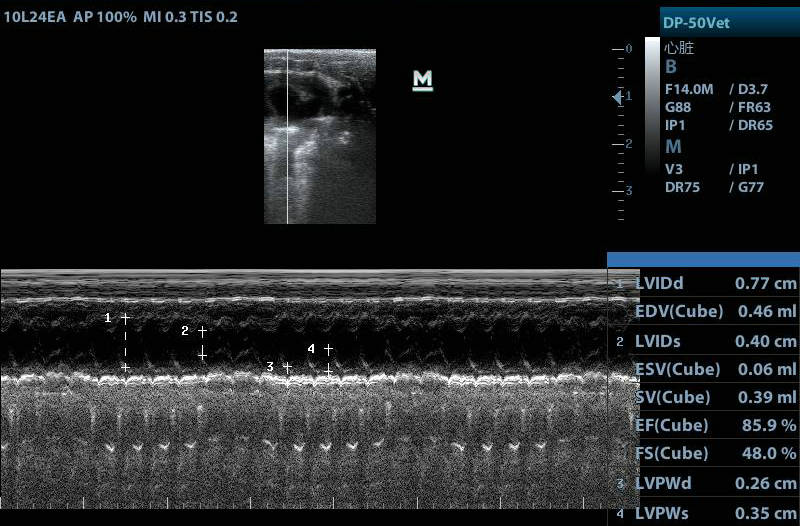

Supplement: Supplemental Information 5 [file peerj-09-10920-s005.zip › data/B │1⁄4/control/A5.JPG]

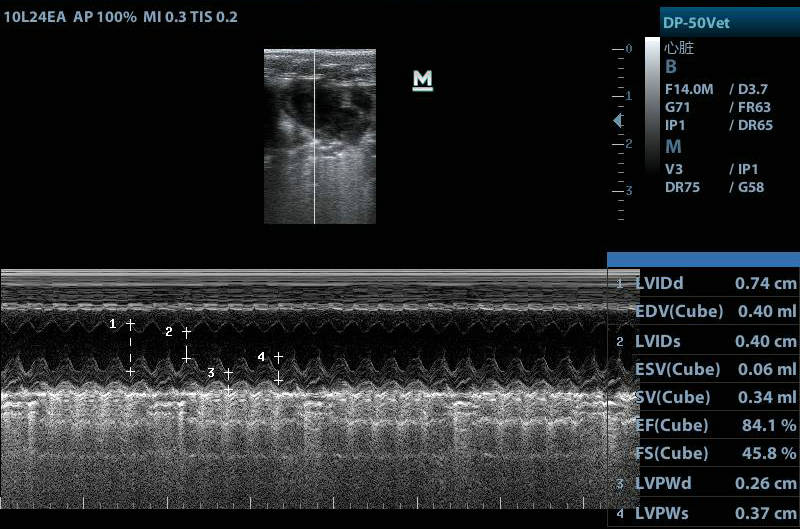

Supplement: Supplemental Information 5 [file peerj-09-10920-s005.zip › data/B │1⁄4/control/A6.JPG]

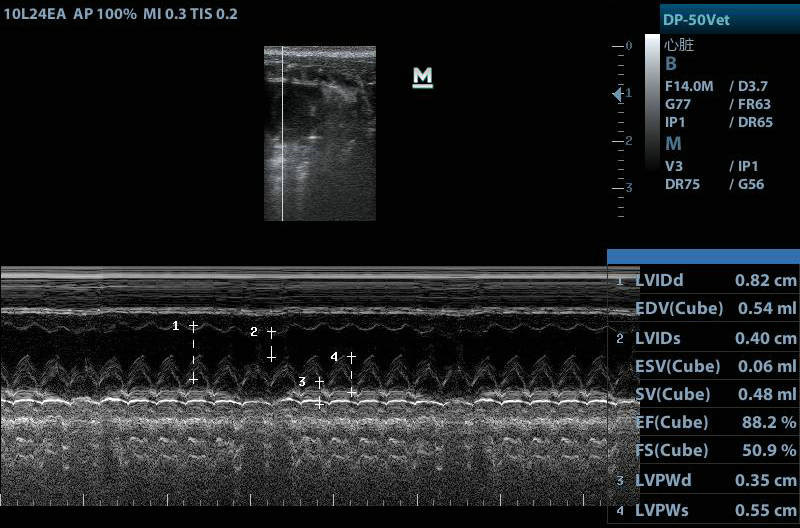

Supplement: Supplemental Information 5 [file peerj-09-10920-s005.zip › data/B │1⁄4/model/B1.JPG]

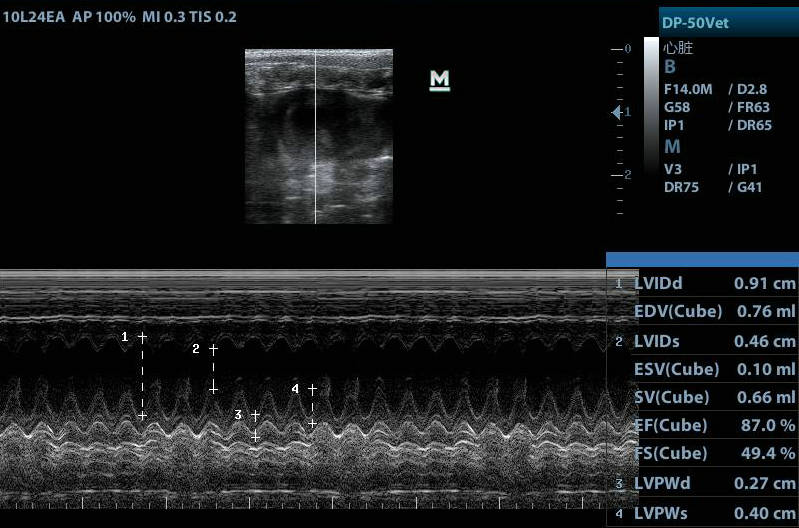

Supplement: Supplemental Information 5 [file peerj-09-10920-s005.zip › data/B │1⁄4/model/B2.JPG]

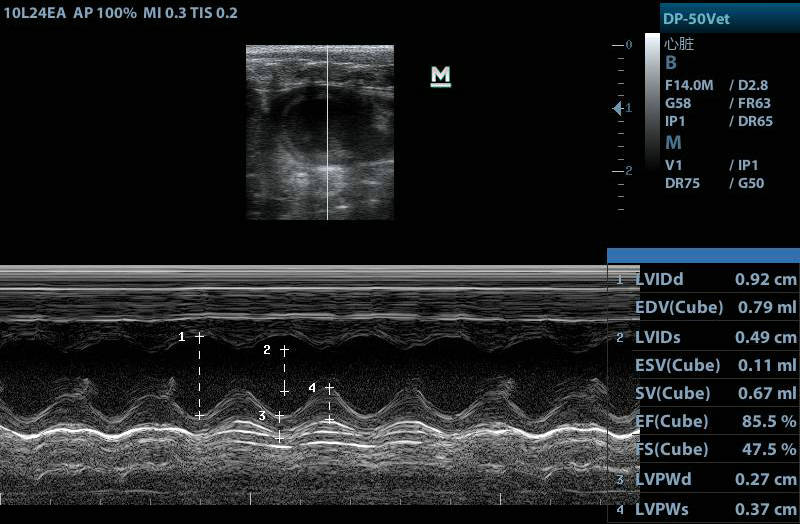

Supplement: Supplemental Information 5 [file peerj-09-10920-s005.zip › data/B │1⁄4/model/B3.JPG]

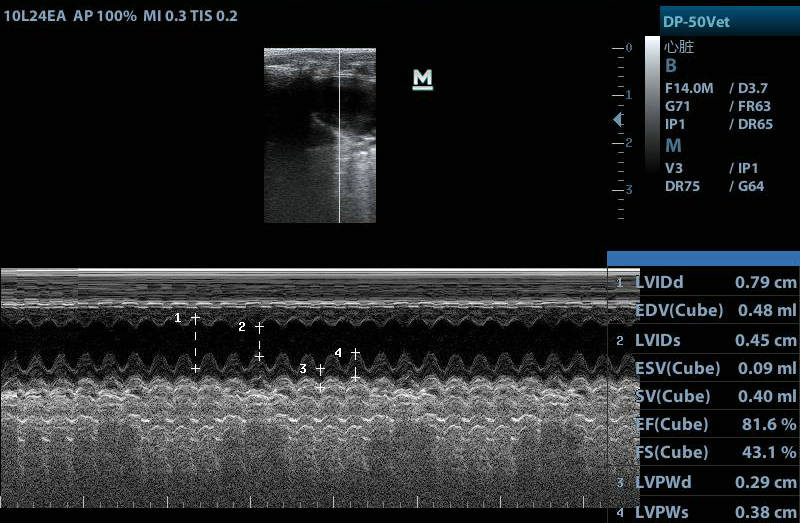

Supplement: Supplemental Information 5 [file peerj-09-10920-s005.zip › data/B │1⁄4/model/B4.JPG]

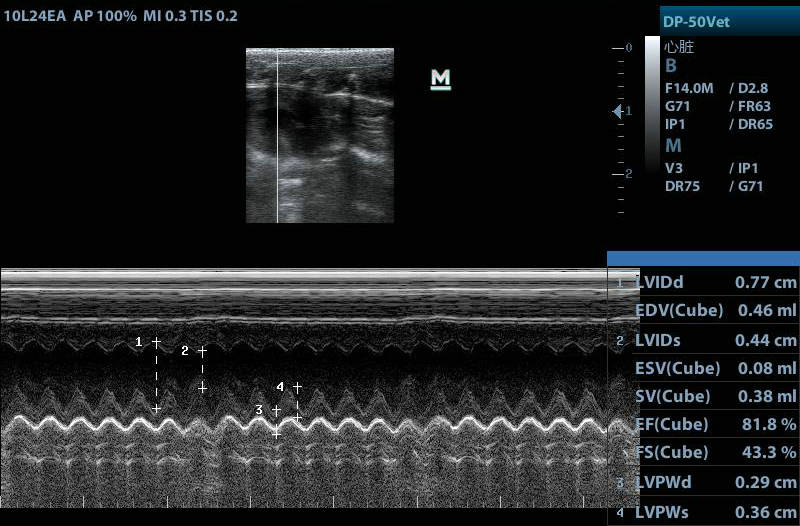

Supplement: Supplemental Information 5 [file peerj-09-10920-s005.zip › data/B │1⁄4/model/B5.JPG]

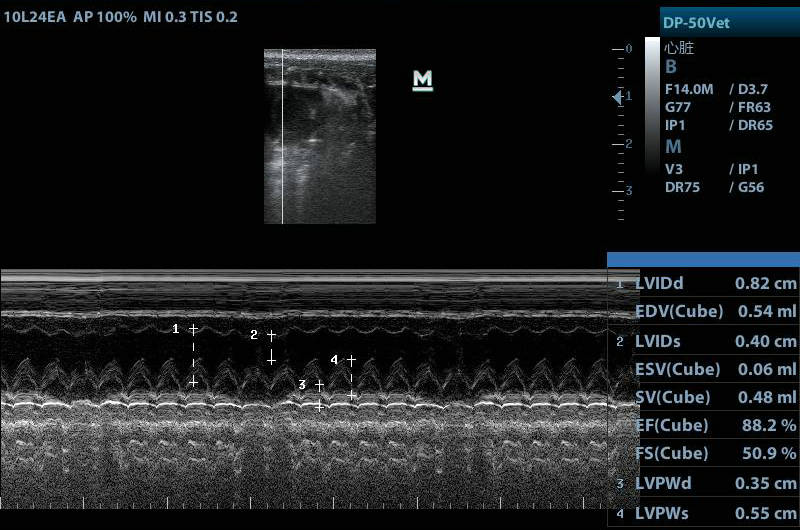

Supplement: Supplemental Information 5 [file peerj-09-10920-s005.zip › data/B │1⁄4/model/B6.JPG]

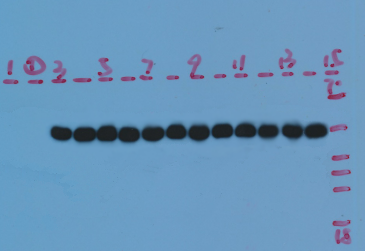

Supplement: Supplemental Information 5 [file peerj-09-10920-s005.zip › data/c and model/GAPDH.tif]

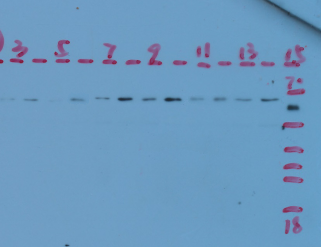

Supplement: Supplemental Information 5 [file peerj-09-10920-s005.zip › data/c and model/P70S6K2.tif]

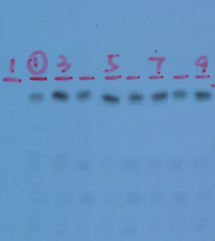

Supplement: Supplemental Information 5 [file peerj-09-10920-s005.zip › data/c and model/p-IRS1 -2.tif]

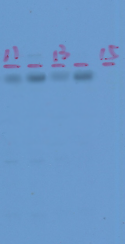

Supplement: Supplemental Information 5 [file peerj-09-10920-s005.zip › data/c and model/p-IRS1.tif]

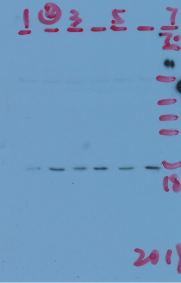

Supplement: Supplemental Information 5 [file peerj-09-10920-s005.zip › data/c and model/RHEB.tif]

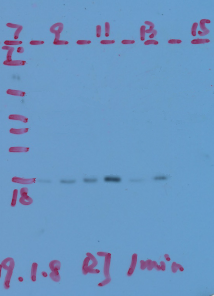

Supplement: Supplemental Information 5 [file peerj-09-10920-s005.zip › data/c and model/RHEB-2.tif]

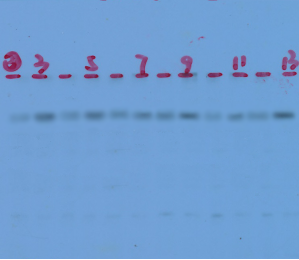

Supplement: Supplemental Information 5 [file peerj-09-10920-s005.zip › data/c and model/Rictor.tif]

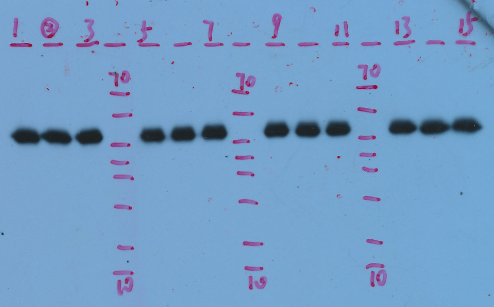

Supplement: Supplemental Information 5 [file peerj-09-10920-s005.zip › data/M MAAV AAVNC/GAPDH -2.tif]

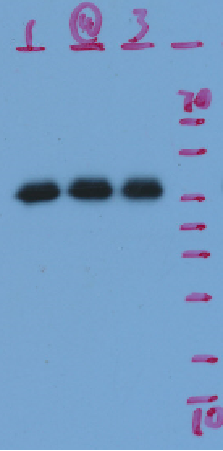

Supplement: Supplemental Information 5 [file peerj-09-10920-s005.zip › data/M MAAV AAVNC/GAPDH -3.tif]

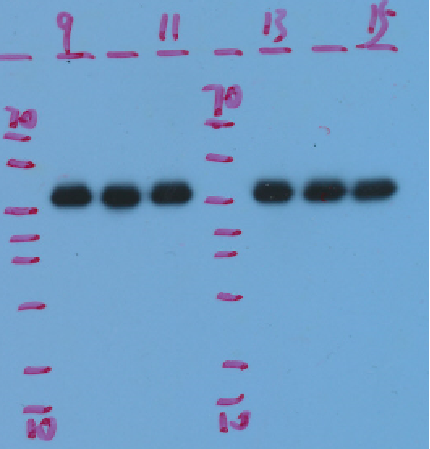

Supplement: Supplemental Information 5 [file peerj-09-10920-s005.zip › data/M MAAV AAVNC/GAPDH -4.tif]

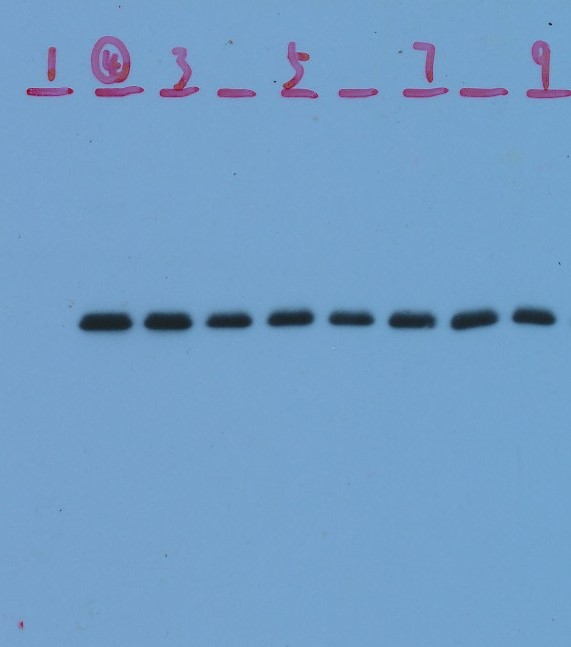

Supplement: Supplemental Information 5 [file peerj-09-10920-s005.zip › data/M MAAV AAVNC/GAPDH.jpg]

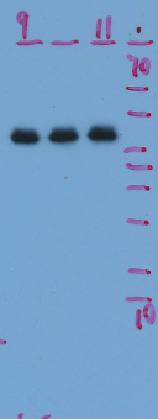

Supplement: Supplemental Information 5 [file peerj-09-10920-s005.zip › data/M MAAV AAVNC/GAPDH.tif]

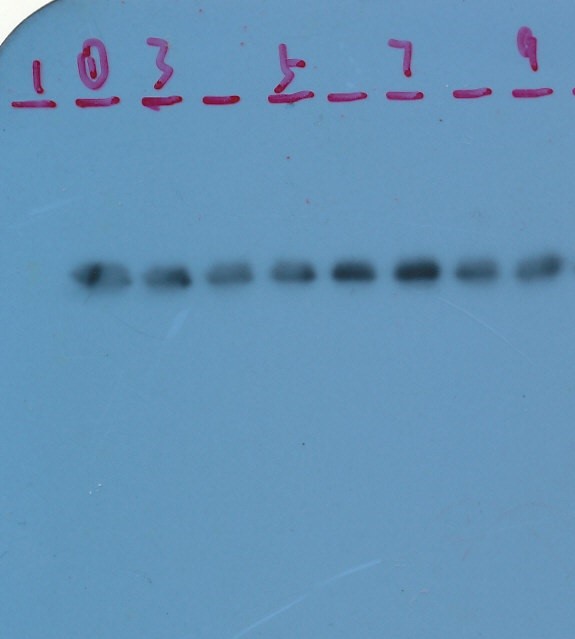

Supplement: Supplemental Information 5 [file peerj-09-10920-s005.zip › data/M MAAV AAVNC/GLUT4.jpg]

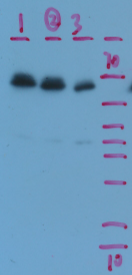

Supplement: Supplemental Information 5 [file peerj-09-10920-s005.zip › data/M MAAV AAVNC/P70S6K2 -2.tif]

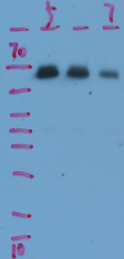

Supplement: Supplemental Information 5 [file peerj-09-10920-s005.zip › data/M MAAV AAVNC/P70S6K2 -3.tif]

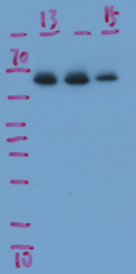

Supplement: Supplemental Information 5 [file peerj-09-10920-s005.zip › data/M MAAV AAVNC/P70S6K2 -4.tif]

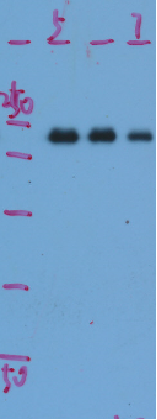

Supplement: Supplemental Information 5 [file peerj-09-10920-s005.zip › data/M MAAV AAVNC/P70S6K2 -5.tif]

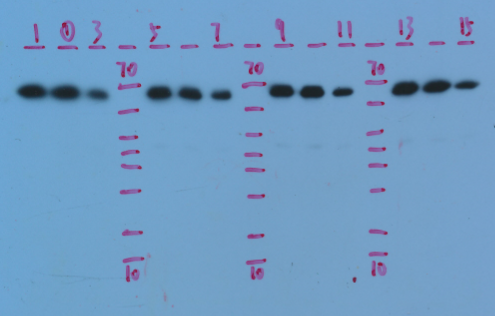

Supplement: Supplemental Information 5 [file peerj-09-10920-s005.zip › data/M MAAV AAVNC/P70S6K2.tif]

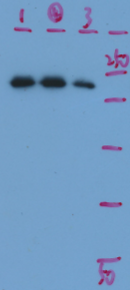

Supplement: Supplemental Information 5 [file peerj-09-10920-s005.zip › data/M MAAV AAVNC/p-IRS1 -2.tif]

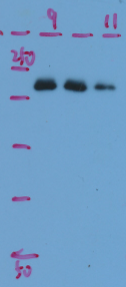

Supplement: Supplemental Information 5 [file peerj-09-10920-s005.zip › data/M MAAV AAVNC/p-IRS1 -3.tif]

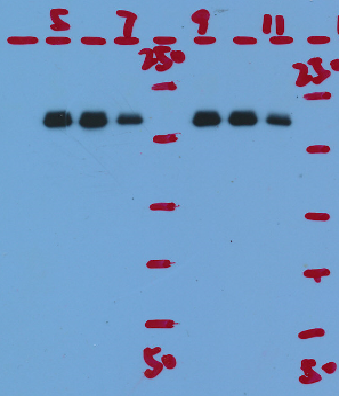

Supplement: Supplemental Information 5 [file peerj-09-10920-s005.zip › data/M MAAV AAVNC/p-IRS1 -4.tif]

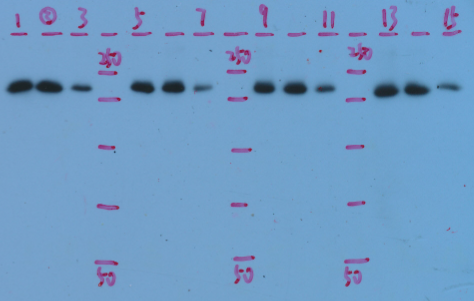

Supplement: Supplemental Information 5 [file peerj-09-10920-s005.zip › data/M MAAV AAVNC/p-IRS1.tif]

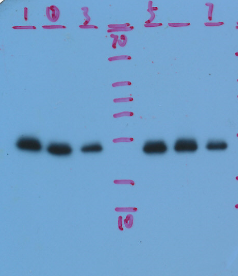

Supplement: Supplemental Information 5 [file peerj-09-10920-s005.zip › data/M MAAV AAVNC/RHEB -2.tif]

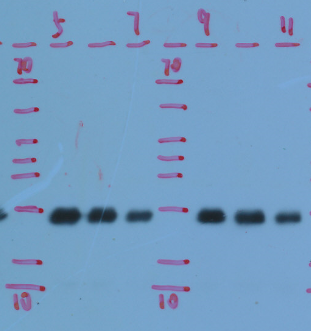

Supplement: Supplemental Information 5 [file peerj-09-10920-s005.zip › data/M MAAV AAVNC/RHEB -3.tif]

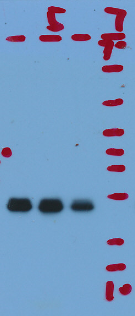

Supplement: Supplemental Information 5 [file peerj-09-10920-s005.zip › data/M MAAV AAVNC/RHEB -4.tif]

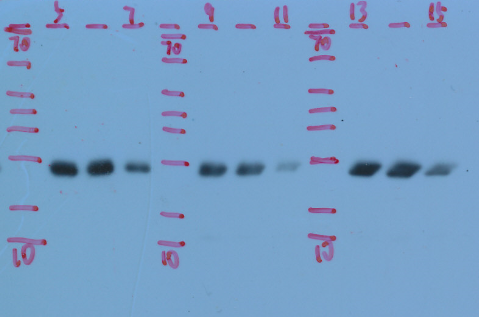

Supplement: Supplemental Information 5 [file peerj-09-10920-s005.zip › data/M MAAV AAVNC/RHEB.tif]

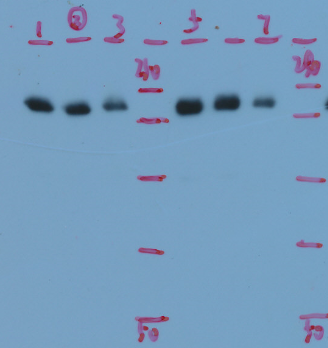

Supplement: Supplemental Information 5 [file peerj-09-10920-s005.zip › data/M MAAV AAVNC/RICHOR 2.tif]

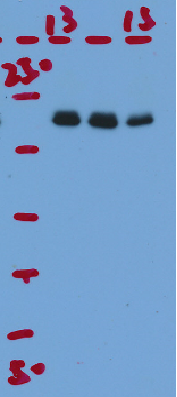

Supplement: Supplemental Information 5 [file peerj-09-10920-s005.zip › data/M MAAV AAVNC/RICHOR -3.tif]

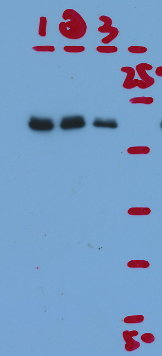

Supplement: Supplemental Information 5 [file peerj-09-10920-s005.zip › data/M MAAV AAVNC/RICHOR -4.tif]

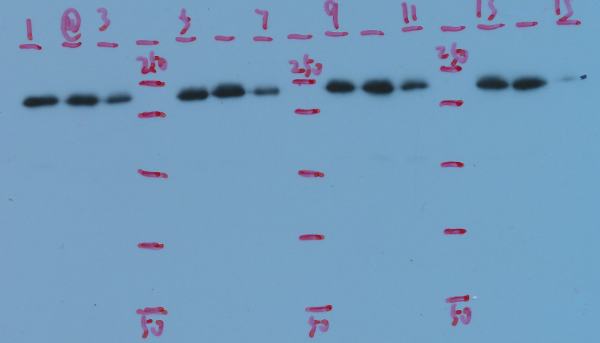

Supplement: Supplemental Information 5 [file peerj-09-10920-s005.zip › data/M MAAV AAVNC/RICHOR.tif]
